# Probiotics for the prevention of antibiotic-associated diarrhea: an umbrella review and second-order meta-analysis of 21 systematic reviews covering 170 unique randomized controlled trials

**DOI:** 10.3389/fnut.2026.1994645

**Published:** 2026-10-27

**Authors:** Fenglin Xi, Caihua Xu, Ping Li, Shenghui Zhou, Jiali Liu, Shangru Zhou, Yajun Wang, Jiaxin Yu

**Affiliations:** 1 Baiyin City Central Hospital, Baiyin, Gansu, China; 2 Evidence-based Medicine Center, School of Basic Medical Sciences, Lanzhou University, Lanzhou, Gansu, China

**Keywords:** antibiotic-associated diarrhea, probiotics, second-order meta-analysis, systematic reviews, umbrella review

## Abstract

**Background:**

Antibiotic-associated diarrhea (AAD) is a common adverse event of antimicrobial therapy. Probiotics have been investigated for the prevention of AAD, but the evidence remains heterogeneous and fragmented. We conducted an umbrella review and second-order meta-analysis to comprehensively evaluate the efficacy and safety of probiotics for AAD prevention.

**Methods:**

We searched PubMed, Embase, Web of Science, the Cochrane Library, the Cochrane Database of Systematic Reviews, CINAHL, CENTRAL, and Scopus from inception to March 21, 2026. Systematic reviews (SRs) with meta analyses (MAs) of randomized controlled trials (RCTs) evaluating oral probiotics for AAD prevention were included. Two reviewers independently screened studies, extracted data, and assessed methodological quality using AMSTAR 2. Overlap of primary studies was quantified with the corrected covered area (CCA) and the GROOVE tool. A second-order meta-analysis of review-level effect estimates was performed using random-effects models, with sensitivity analyses addressing review quality and primary-study overlap. Reporting followed PRIOR and PRISMA 2020.

**Results:**

Twenty-one SRs/MAs were included; together they contained 478 RCTs, corresponding to 170 unique RCTs. Most SRs/MAs were rated low or critically low quality by AMSTAR 2. The CCA indicated moderate overall overlap (9.06%), but 71 of 210 pairs of reviews showed very high overlap (≥15%). In the pooled analysis, probiotics were associated with a lower risk of AAD (RR 0.54, 95% CI 0.48–0.61; OR 0.37, 95% CI 0.31–0.46). Because the SRs/MAs share many primary trials, these estimates are probably more precise than the evidence warrants. Estimates were similar in leave-one-review-out analyses (RR 0.54–0.55) and when restricted to SRs/MAs rated high or low quality (RR 0.52, 95% CI 0.48–0.57), but weaker and less precise in subsets of SRs/MAs with low pairwise overlap (RR 0.59–0.63). Pooled estimates were similar in children (RR 0.46) and adults (RR 0.55), whereas in the four SRs/MAs reporting on older adults no clear benefit was seen (RR 0.96, 95% CI 0.85–1.08). In an exploratory comparison based on three overlapping SRs/MAs, the estimate was lower for daily doses ≥5 × 10^9^ CFU than for lower doses; this comparison is confounded by age and product and cannot be applied to individual products. No increase in adverse events was detected, but only two SRs/MAs reported this outcome.

**Conclusion:**

Across published SRs/MAs, probiotics were associated with a lower risk of antibiotic-associated diarrhea, without evidence of increased adverse events. However, only 1 of 21 SRs/MAs was rated high quality and 13 were rated critically low, the SRs/MAs share many primary trials, and the interventions were heterogeneous in strain, dose, and formulation. The findings therefore represent low-certainty evidence, do not show that any specific probiotic product is effective, and should not be used to support class-wide or product-specific claims. High-quality, strain-specific RCTs and well-conducted, registered SRs/MAs are needed.

**Systematic review registration:**

https://www.crd.york.ac.uk/PROSPERO/view/CRD420261404660, identifier PROSPERO (CRD420261404660).

## 1 Introduction

Antibiotic-associated diarrhea (AAD) is among the most frequent adverse events related to antibiotic exposure. It is commonly defined as the passage of at least three soft or liquid stools per day persisting for at least 24 h during antibiotic treatment or within 2 months after cessation, in the absence of another identifiable cause (1). The reported incidence of AAD varies substantially across studies, populations, and antibiotic classes, but is commonly estimated to range from approximately 5–35%, especially in hospitalized patients compared to outpatients (2). Although most cases are mild and self-limited, AAD can prolong hospitalization, reduce adherence to antibiotic treatment, increase healthcare costs, and in some patients progress to severe colitis or *clostridium difficile* infection (3, 4).

The disruption of gut microbiota caused by antibiotics is a key mechanism underlying AAD. Antibiotic exposure reduces microbial diversity, alters metabolic function, and weakens colonization resistance, thereby facilitating overgrowth of opportunistic pathogens and impairing intestinal homeostasis (5). Additional mechanisms, including altered bile acid metabolism, mucosal barrier dysfunction, and immune dysregulation, may also contribute to symptom development (6). Given this pathophysiology, interventions that preserve or restore the gut microbiome have attracted considerable interest for AAD prevention.

Probiotics are defined as live microorganisms that, when administered in adequate amounts, confer a health benefit on the host (7). Experimental and clinical studies suggest that probiotics may reduce AAD risk by restoring microbial balance, strengthening epithelial barrier function, competing with pathogenic organisms, and modulating host immune response (8–10).

Over the past two decades, numerous randomized controlled trials (RCTs) and systematic reviews (SRs) and meta analyses (MAs) have evaluated the role of probiotics in preventing AAD. However, conclusions have varied because of differences in probiotic strains, dosages, treatment duration, patient populations, and outcome definitions. Probiotic products also differ in product quality: strain identity, viable cell counts, and microbial purity may deviate from label claims, while clinical effects are strain-specific and should not be assumed to apply to other strains or formulations (4). In addition, the methodological quality of existing reviews and the extent of overlap among primary studies have not been comprehensively assessed.

An umbrella review can address these limitations by synthesizing the totality of evidence from published SRs/MAs while evaluating their methodological quality, potential redundancy, and consistency of findings. Therefore, we conducted an umbrella review with a quantitative second-order meta-analysis (meta-meta-analysis) of review-level effect estimates to assess the efficacy and safety of probiotics for preventing AAD, to explore potential effect modifiers, and to appraise the credibility of the current evidence base. Specifically, we aimed to: ① systematically summarize the available meta-analytic evidence, ② quantitatively synthesize review-level effect estimates using a second-order meta-analysis, ③ evaluate the methodological quality of included SRs/MAs using A MeaSurement Tool to Assess Systematic Reviews (AMSTAR-2), ④ quantify primary-study overlap across SRs/MAs and examine its influence on the pooled estimates, and ⑤ explore potential effect modifiers through subgroup analyses.

## 2 Methods

### 2.1 Study design and protocol registration

This umbrella review was reported in accordance with the Preferred Reporting Items for Overviews of Reviews (PRIOR) statement (11), with PRISMA 2020 additionally consulted for general systematic-review reporting items (12). Completed PRIOR and PRISMA 2020 checklists are provided in Supplementary Tables S1, S2, respectively. The protocol was prospectively registered in PROSPERO (CRD420261404660).

### 2.2 Search strategy

We systematically searched PubMed, Embase, Web of Science, the Cochrane Library, the Cochrane Database of Systematic Reviews, CINAHL, CENTRAL, and Scopus from inception to March 21, 2026. The search strategy combined MeSH and free-text keywords related to probiotics, AAD, systematic review, and meta-analysis. The detailed search strategy for each database is provided in Supplementary Table S3. References of relevant articles were also screened manually.

### 2.3 Eligibility criteria

All records were imported into EndNote X9.3.3 for de-duplication. Two reviewers independently screened titles, abstracts, and full-text articles for eligibility. Discrepancies were resolved through discussion, with arbitration by a third reviewer when necessary. When full texts were unavailable or reporting was unclear, corresponding authors were contacted for clarification.

Eligibility criteria were defined according to the PICOS framework. We included SRs/MAs involving participants of any age who received oral antibiotic therapy for any clinical indication. Studies explicitly enrolling individuals with pre-existing diarrhea at baseline or laxative-induced diarrhea were excluded to reduce confounding in the evaluation of probiotic efficacy.

Eligible interventions were defined as orally administered probiotics, delivered as supplements (e.g., capsules, sachets) or probiotic-containing foods (e.g., yogurt), during or after antibiotic exposure, regardless of formulation, duration, or dose. SRs/MAs evaluating probiotics in combination with other therapies were excluded unless the independent effect of probiotics could be clearly distinguished.

We included peer-reviewed SRs with MAs of RCTs comparing probiotics with placebo, no intervention, or another active control, provided that AAD incidence was reported as a primary or major outcome. For this overview, a systematic review was defined as a review that (i) addressed a clearly stated research question with prespecified inclusion criteria; (ii) searched at least two bibliographic databases and reported the search terms or search strategy; (iii) described how eligible studies were selected; and (iv) synthesized the results of the included studies quantitatively in a meta-analysis. Reviews that did not meet this definition, for example narrative reviews or reviews without a reproducible search, were excluded. Studies were also excluded if they did not report extractable effect estimates for diarrhea incidence (e.g., relative risk (RR) or odds ratio (OR) with 95% confidence intervals (CIs)), or if they were duplicate publications, conference abstracts, or non-clinical studies (e.g., animal or *in vitro* studies).

### 2.4 Data extraction and quality assessment

Two reviewers independently extracted data using a standardized form. Extracted information included the first author, publication year, number of included RCTs, total sample size, participant characteristics, probiotic type, comparator, pooled effect estimate, 95% CIs, heterogeneity statistics, and risk-of-bias assessment tool used in the original study. Data were independently verified by two additional reviewers to ensure accuracy.

Methodological quality of the included SRs/MAs was assessed using the AMSTAR-2 (13). This instrument evaluates 16 domains, including seven critical domains. Overall confidence in the results of each SR/MA was categorized as high, moderate, low, or critically low according to the AMSTAR 2 guidance. Two reviewers performed the assessment independently, and discrepancies were resolved by consensus. Each item was systematically rated as “Yes,” “Partial Yes,” or “No” according to established criteria. The following items are considered critical: protocol registration, adequacy of search strategy, justification for excluding individual studies, risk of bias assessment, appropriateness of meta-analysis methods, use of risk of bias during interpretation and assessment of publication bias.

### 2.5 Assessment and handling of overlap

To evaluate the extent of primary study redundancy across the included SRs/MAs, we utilized the Graphical Representation of Overlap for OVerviews (GROOVE) tool (14). The overall degree of overlap was quantified using the Corrected Covered Area (CCA), calculated as CCA = (N-r)/(rc-r), where N represents the total number of primary studies (including double counting), *r* the number of unique index publications, and *c* the number of SRs/MAs. In accordance with Pieper’s criteria, the degree of overlap was categorized as slight (<5%), moderate (5% to <10%), high (10% to <15%), or very high (≥15%) (15). To enhance the transparency and interpretability of these findings, we employed the GROOVE tool to generate a graphical evidence map (14). By constructing a matrix of primary studies against SRs/MAs, the GROOVE tool facilitated the calculation of pairwise overlap and produced a color-coded matrix, visually distinguishing areas of high evidence density from those with unique research contributions.

Overlap was not removed from the primary analysis. All 21 SRs/MAs met the eligibility criteria and differ in populations, search dates, and inclusion criteria, so the primary pooled estimates describe the whole body of published SRs/MAs. Because the same RCT can contribute to several SRs/MAs, these estimates are not independent, and their confidence intervals should be regarded as too narrow. To examine how overlap and SRs/MAs quality influenced the pooled estimates, we carried out sensitivity analyses that were not specified in the protocol and were added during peer review: (i) leave-one-review-out analysis; (ii) restriction to SRs/MAs rated high or low quality by AMSTAR 2; and (iii) analysis of subsets of SRs/MAs with low overlap. These subsets were built by ranking the RR-reporting SRs/MAs by AMSTAR 2 rating, then by number of RCTs, then by publication year (latest first), and including a SR/MA only if its pairwise CCA with every SR/MA already included was below 15% (or below 10%).

### 2.6 Statistical analysis

All analyses were performed using STATA 17 (StataCorp LLC, College Station, TX, United States). Review-level effect estimates (RR, OR, or risk difference) were extracted from each included MA (Supplementary Table S4), log-transformed where applicable, with standard errors derived from the reported 95%CIs, and pooled separately by effect measure using a DerSimonian-Laird random-effects model.

Heterogeneity was assessed using the *Cochran Q*-test and quantified with *I*^2^ statistic. *I*^2^ values of 25, 50, and 75% represented low, moderate, and high heterogeneity, respectively. Prespecified subgroup analyses examined potential effect modification by age group, clinical setting, number of probiotic strains, probiotic dose, and risk of bias. Subgroup analyses used the stratum-specific estimates reported within the included SRs/MAs, so a SR/MA could contribute more than one estimate to a stratum and could contribute to more than one stratum; the number of contributing SRs/MAs is given for each analysis. Subgroup comparisons are therefore comparisons across SRs/MAs and are exploratory. Older adults were defined as persons older than 65 years. Publication bias was assessed at the level of the included SRs/MAs through funnel plot inspection and Egger’s regression test. A two-sided *p*-value below 0.05 was used as the threshold for statistical testing; effect sizes and 95%CIs are reported so that the relevance of an effect can be judged separately from its *p*-value.

## 3 Results

### 3.1 Study selection

The database search yielded 4,774 records, of which 2,421 remained after duplicate removal. Following title and abstract screening, 2,297 records were excluded. Full-text assessment of the remaining records led to the exclusion of additional non-eligible articles. Ultimately, 21 SRs with MAs met the inclusion criteria and were included in this umbrella review (16–36). The selection process is shown in the PRISMA flow diagram (Figure 1). A list of articles excluded at the full text screening stage with reasons for exclusion can be found in Supplementary Table S5. Collectively, the included reviews contained 478 trial entries, which correspond to 170 unique RCTs. The summed sample size of the reviews (101,416) counts trials that appear in several SRs/MAs repeatedly and therefore does not represent the number of unique participants.

**Figure 1:**
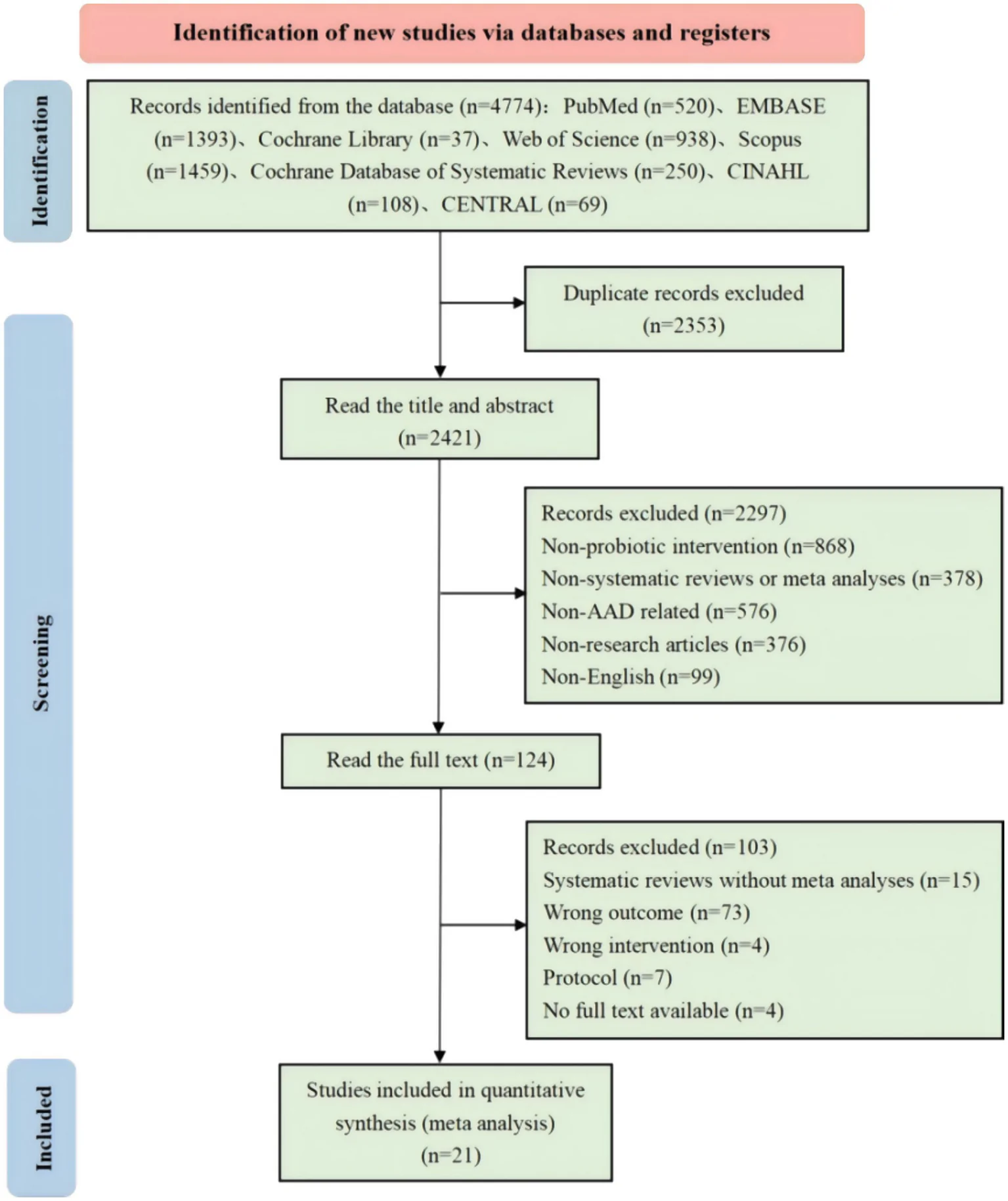
PRISMA flow diagram.

### 3.2 Characteristics of the included SRs/MAs

The characteristics of the included SRs/MAs are summarized in Table 1. Eligible SRs/MAs were published between 2002 and 2025. The number of RCTs per SRs/MAs ranged from 6 to 63, and total sample sizes ranged from 707 to 11,811 participants. 5 SRs/MAs focused on adults (22, 25, 27, 33, 36), 5 on children (18, 20, 29, 31, 35), one on older adults (34), and 10 included mixed-age populations (16, 17, 19, 21, 23, 24, 26, 28, 30, 32). The most frequently investigated probiotic genera were Lactobacillus, Bifidobacterium, and Saccharomyces, either alone or in combination. Other genera included Streptococcus, Enterococcus, Bacillus, Clostridium, Lactococcus, and *Leuconostoc cremoris*. All SRs/MAs used placebo as the comparator. 18 SRs/MAs evaluated multi-strain or mixed probiotic formulations, whereas 3 focused on single-strain interventions. Risk-of-bias tools used in the original SRs/MAs varied, with the Cochrane Risk of Bias tool being the most common. The primary outcome across all SRs/MAs was AAD incidence, and 4 SRs/MAs reported adverse events, including 2 using RD (28, 31), 1 using OR (30), and 1 using RR (33). In Table 1, interventions could be characterized only at genus level; strain identity, formulation, and product quality were not evaluated in this umbrella review.

**Table 1:** Characteristics of the included SRs/MAs.

Study ID	No. of included RCTs	Country	Target population	Sample size	Intervention		Outcomes	Risk assessment tool
					Experimental group	Control group		
Cremonini et al. (16)	7	Italy	All ages	881	*Lactobacillus* spp., *Saccharomyces* spp.	Placebo	Incidence of AAD	NR
D’Souza et al. (17)	9	UK	All ages	1,214	*Saccharomyces* spp., *Lactobacillus* spp., *Enterococci* spp.	Placebo	Incidence of AAD	NR
Johnston et al. (18)	6	Canada	Children	707	*Lactobacillus* spp., *Bifidobacterium* spp., *Saccharomyces* spp.	Placebo	Incidence of AAD	Jadad scale
McFarland (19)	25	USA	All ages	2,810	*Saccharomyces* spp., *Lactobacillus* spp.	Placebo	Incidence of AAD	Scale reported by the U. S. Preventive Services Task Force
Szajewska et al. (20)	6	Poland	Children	766	*Lactobacillus* spp., *Saccharomyces* spp.	Placebo	Incidence of AAD	Standard criteria
Kale-Pradhan et al. (21)	10	USA	All ages	1862	*Lactobacillus* spp.	Placebo	Incidence of AAD	Jadad scale
Avadhani and Miley (22)	8	USA	Adults	1,220	*Lactobacillus* spp., *Streptococcus* spp., *Saccharomyces* spp., *Bifidobacterium* spp.	Placebo	Incidence of AAD	JBI
Hempel et al. (23)	62	USA	All ages	11,811	*Lactobacillus* spp., *Bifidobacterium* spp., *Saccharomyces* spp., *Streptococcus* spp., *Enterococcus* spp., *Bacillus* spp.	Placebo	Incidence of AAD	Cochrane Risk of Bias tool
Videlock and Cremonini (24)	34	USA	All ages	4,138	*Lactobacillus* spp., *Bifidobacterium* spp., *Enterococcus* spp., *Streptococcus* spp., *Saccharomyces* spp.	Placebo	Incidence of AAD	Cochrane Handbook for systematic reviews of Interventions
Pattani et al. (25)	16	Canada	Adults	2,296	*Lactobacillus* spp., *Saccharomyces* spp., *Enterococcus* spp.	Placebo	Incidence of AAD	Scale reported by the U. S. Preventive Services Task Force
Szajewska and Kołodziej (26)	21	Poland	All ages	4,780	*Saccharomyces* spp.	Placebo	Incidence of AAD	Cochrane Collaboration’s tool
Jafarnejad et al. (27)	30	Iran	Adults	7,260	*Lactobacillus* spp., *Bifidobacterium* spp., *Enterococcus* spp., *Streptococcus* spp., *Saccharomyces* spp.	Placebo	Incidence of AAD	Cochrane Risk of Bias tool
Blaabjerg et al. (28)	17	Denmark	All ages	3,631	*Lactobacillus* spp., *Lactococcus* spp., *Bacillus* spp., *Bifidobacterium* spp., *Saccharomyces* spp., *Leuconostoc cremoris*, *Clostridium* spp., *Streptococcus* spp.	Placebo	Incidence of AAD, adverse events	Cochrane Collaboration’s tool
Xu et al. (29)	30	China	Children	7,225	*Bifidobacterium* spp., *Lactobacillus* spp., *Enterococcus* spp., *Bacillus* spp., *Streptococcus* spp., *Clostridium* spp.	Placebo	Incidence of AAD	Jadad scale
Cai et al. (30)	51	China	All ages	9,569	*Lactobacillus* spp., *Bacillus* spp., *Saccharomyces* spp., *Bifidobacterium* spp., *Streptococcus* spp., *Clostridium* spp.	Placebo	Incidence of AAD, adverse events	Cochrane Collaboration’s tool
Guo et al. (31)	33	China	Children	6,352	*Lactobacillus* spp., *Bifidobacterium* spp., *Streptococcus* spp., *Saccharomyces* spp.	Placebo	Incidence of AAD, adverse events	Cochrane Risk of Bias tool
Goodman et al. (32)	42	Australia	All ages	11,305	*Lactobacillus* spp., *Saccharomyces* spp., *Bifidobacterium* spp., *Bacillus* spp., *Clostridium* spp.	Placebo	Incidence of AAD	Cochrane Risk of Bias tool
Liao et al. (33)	36	China	Adults	9,312	*Lactobacillus* spp., *Saccharomyces* spp., *Bifidobacterium* spp., *Bacillus* spp.	Placebo	Incidence of AAD, adverse events	Cochrane Handbook for systematic reviews of Interventions
Zhang et al. (34)	8	China	Older adults	4,691	*Saccharomyces* spp., *Lactobacillus* spp., *Bifidobacterium* spp.	Placebo	Incidence of AAD	Cochrane Risk of Bias tool
Fadin et al. (35)	12	Brazil	Children	2,159	*Lactobacillus* spp.	Placebo	Incidence of AAD	PEDro scale
Wanyama et al. (36)	15	UK	Adults	7,427	*Lactobacillus* spp., *Bifidobacterium* spp., *Saccharomyces* spp.	Placebo	Incidence of AAD	Cochrane Risk of Bias tool

AAD, Antibiotic-associated diarrhea; NR, no report.

### 3.3 Methodological quality of the included SRs/MAs

According to AMSTAR 2, the overall methodological quality of the included SRs/MAs was limited. Only one SR/MA was rated as high quality (23), whereas 7 were rated as low quality (20, 24, 26–28, 30, 31) and 13 as critically low quality (16–19, 21, 22, 25, 29, 32–36). The most common critical weakness was the absence of a prior protocol or explicit statement that methods were established before the review was conducted. In addition, many SRs/MAs failed to provide a list of excluded studies with justification. In contrast, most SRs/MAs appropriately assessed the risk of bias in the included RCTs. A detailed summary of AMSTAR 2 ratings is presented in Figure 2. Item-level ratings for each SR/MA are provided in Supplementary Table S6.

**Figure 2:**
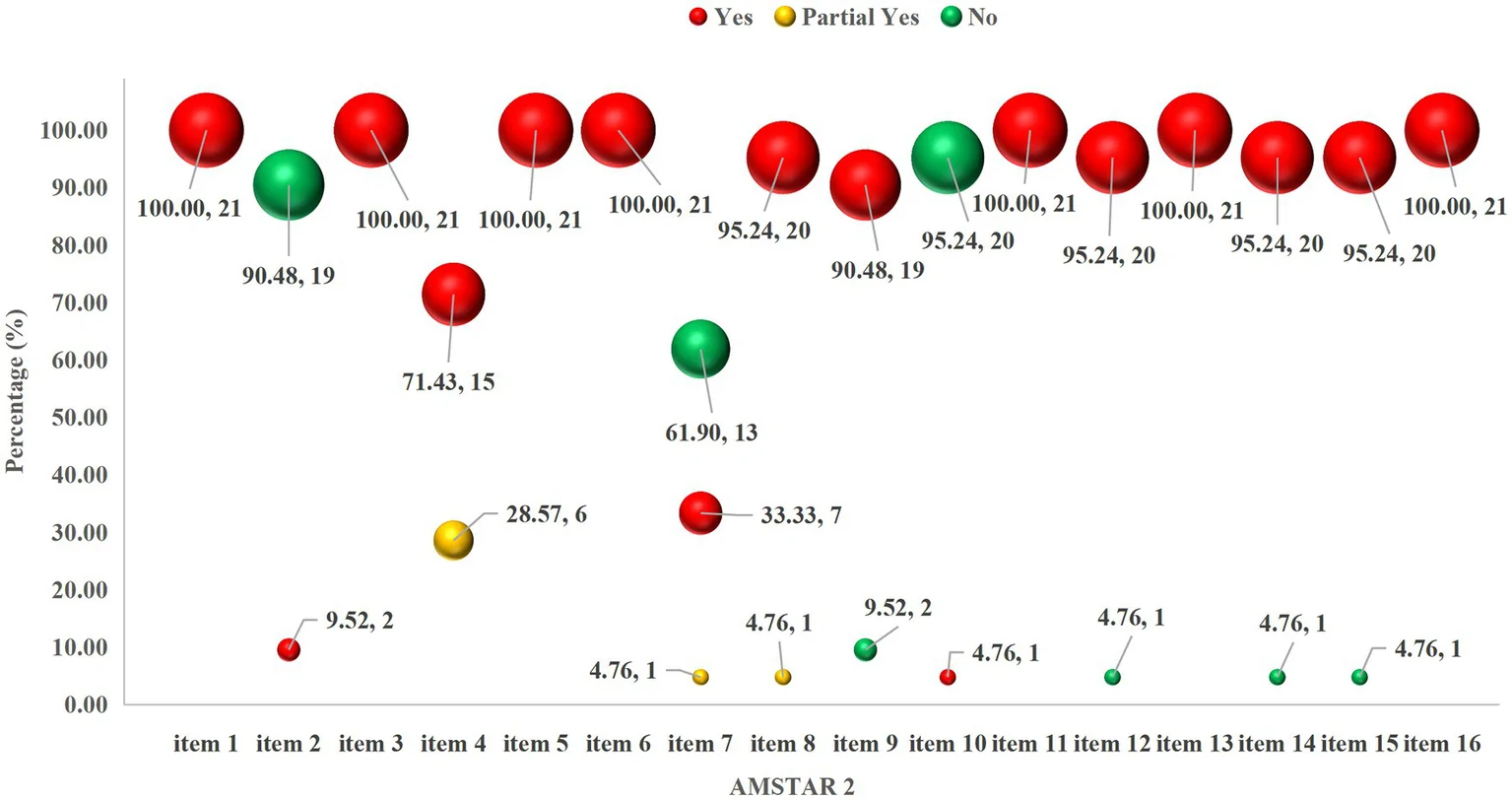
Summary of methodological quality assessment for the included SRs/MAs. (Each bubble shows the proportion of the 21 SRs/MAs (y-axis) that received a given rating (red, yes; yellow, partial yes; green, no) for one of the 16 AMSTAR 2 items (x-axis). Bubble size is proportional to that proportion. The two numbers beside each bubble are the percentage and the number of SRs/MAs with that rating. Critical items are 2, 4, 7, 9, 11, 13, and 15).

### 3.4 Overlap of RCTs across SRs/MAs

The included SRs/MAs listed 478 trial entries in total; after removing duplicates, 170 unique RCTs were identified, indicating substantial overlap across SRs/MAs. The analysis yielded an overall CCA of 9.06% (Figure 3a). The primary-study by SRs/MAs matrix used for the overlap analysis is provided in Supplementary Table S7. Based on Pieper’s criteria, this value falls within the 5% to <10% range, indicating a moderate degree of overlap across the included evidence. The GROOVE matrix further illustrates the distribution of redundancy: of the 210 total pairs, 71 pairs exhibited “very high” overlap (≥15%), 33 “high,” 33 “moderate,” and 73 “slight” overlap, suggesting that while the aggregate overlap is moderate, substantial evidence duplication exists between specific review pairs (Figure 3b). Of the 170 unique RCTs, 88 were included in a single SR/MA and 82 (48.2%) in two or more SRs/MAs (up to 12 SRs/MAs).

**Figure 3:**
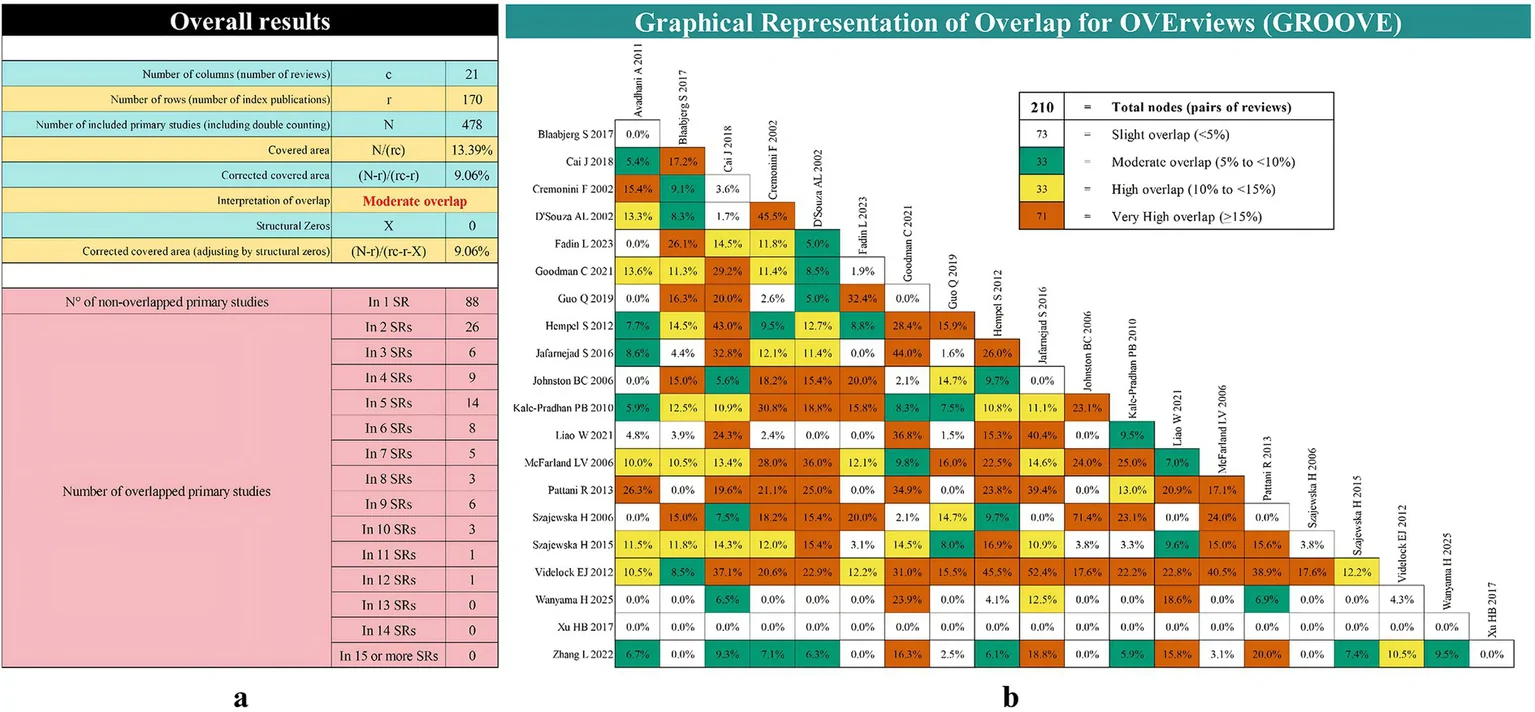
Overlap analysis of primary studies among the included SRs/MAs. **(a)** Overall overlap statistics from the GROOVE tool: *C*, number of SRs/MAs (21); *R*, number of unique primary studies (170); *N*, number of primary studies including double counting (478); Covered area, *N*/(*rc*); Corrected covered area (*CCA*), (*N* − *r*)/(*rc* − *r*) = 9.06%, interpreted as moderate overlap. The lower block shows how many unique RCTs were included in one, two, or more SRs/MAs. **(b)** GROOVE heatmap: Each cell gives the pairwise *CCA* for one pair of SRs/MAs (210 pairs); color indicates the overlap category (white, slight, <5%, 73 pairs; green, moderate, 5% to <10%, 33 pairs; yellow, high, 10% to <15%, 33 pairs; orange, very high, ≥15%, 71 pairs). Higher values mean that two SRs/MAs include many of the same trials.

### 3.5 Effects of probiotics on AAD

#### 3.5.1 Overall analysis

18 SRs/MAs reported effect estimate as RR. The pooled random-effects estimate showed that probiotics reduced the risk of AAD (RR 0.54, 95% CI 0.48–0.61, *p* < 0.001), corresponding to a 46% relative risk reduction (Figure 4a). Nearly all SRs/MAs favored probiotics, except one SR/MA whose estimate was close to the null (34). Heterogeneity was substantial (*I*^2^ = 77.8%, *p* < 0.001).

**Figure 4:**
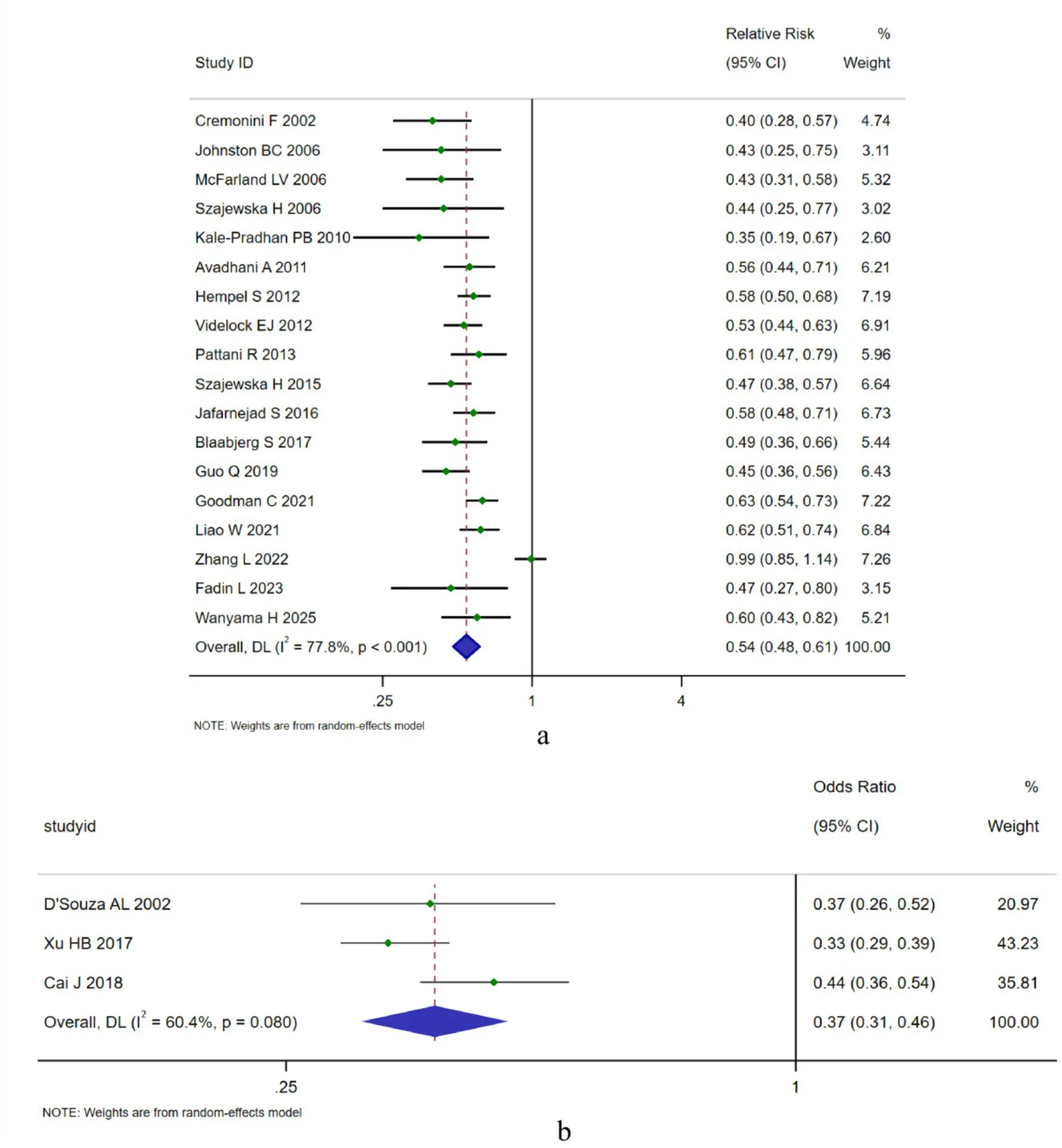
Pooled effect of probiotics on AAD. **(a)** RR. **(b)** OR.

3 SRs/MAs utilized the OR as the effect measure (17, 29, 30) (Figure 4b). The pooled OR also favored probiotics (OR 0.37, 95% CI 0.31–0.46, *p* < 0.001), with moderate heterogeneity (*I*^2^ = 60.4%, *p* = 0.080). However, because only 3 SRs/MAs contributed to this analysis, the heterogeneity estimate should be interpreted cautiously.

#### 3.5.2 Subgroup analysis

To explore potential sources of heterogeneity, we performed subgroup analyses by age group, clinical setting (inpatient vs. outpatient), number of probiotic strains (single vs. mixture), probiotic dose (<5 × 10^9^ vs. ≥5 × 10^9^ CFU/day), and risk of bias (high, low, low/unclear, unclear). The findings are summarized below and presented in Figures 5a–e. The numbers of contributing SRs/MAs were 11 (adults), 8 (children), and 4 (older adults) for age; 2 for clinical setting; 4 for number of strains; 3 for dose; and 6 for risk of bias. Several SRs/MAs contributed more than one estimate to the same stratum, and estimates from SRs/MAs that share trials are not independent.

**Figure 5:**
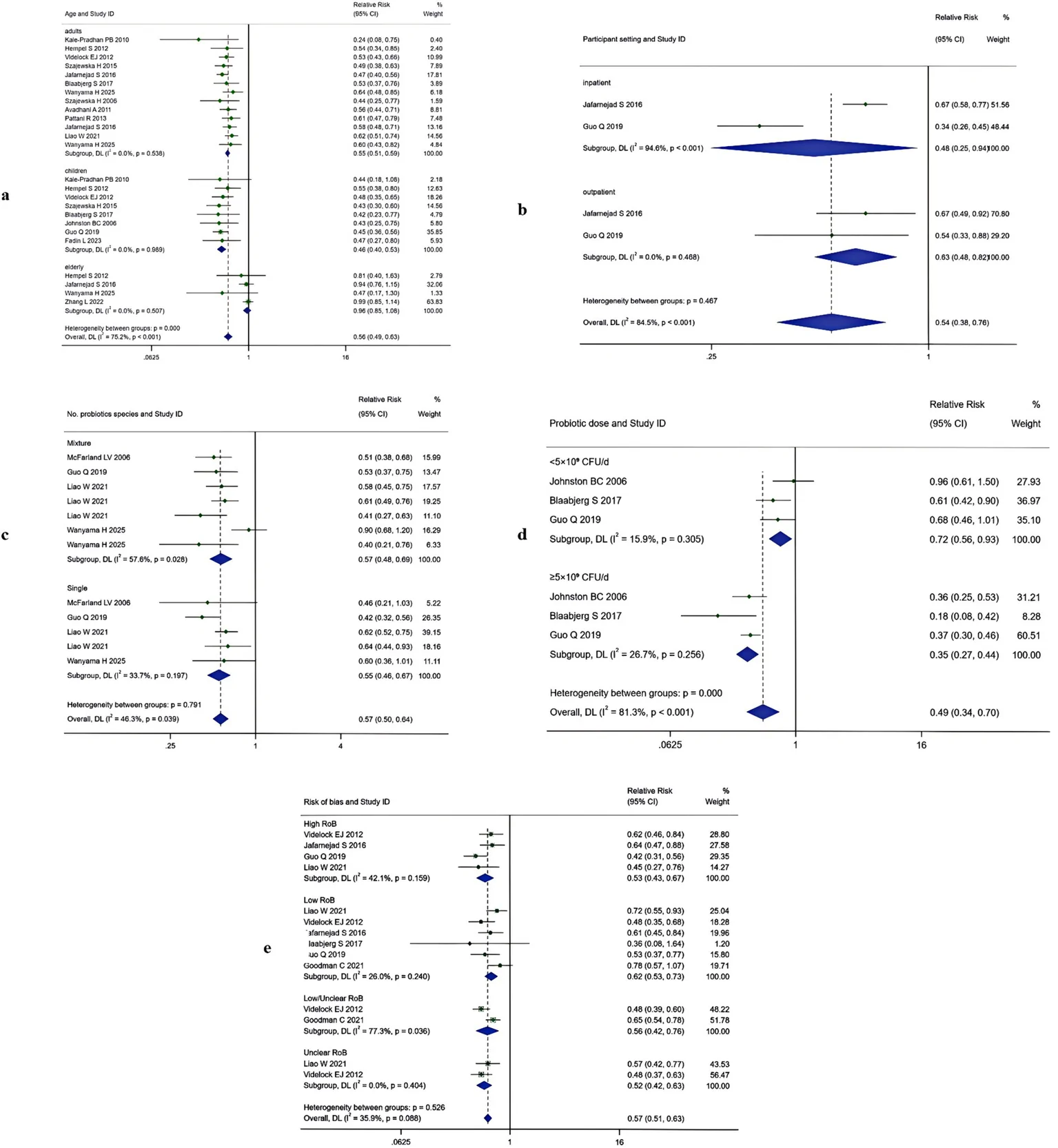
Subgroup analyses. **(a)** Age group, **(b)** clinical setting, **(c)** number of probiotic strains, **(d)** probiotic dose, and **(e)** risk of bias (RCTs in the included SRs/MAs).

##### 3.5.2.1 Age group

Subgroup analyses suggested that the effect of probiotics may vary by age. Probiotics were associated with a lower AAD risk in both children (RR 0.46, 95% CI 0.40–0.53, *p* < 0.001) and adults (RR 0.55, 95% CI 0.51–0.59, *p* < 0.001), whereas no clear protective effect was seen in older adults, defined as persons older than 65 years (RR 0.96, 95% CI 0.85–1.08). Heterogeneity within these subgroups was low. The test for subgroup differences gave *p* < 0.001. Because this comparison is made across reviews, the older-adult stratum is dominated by a single review, and the strata contain overlapping trials, we regard age as a possible effect modifier that requires confirmation and not as an established one.

##### 3.5.2.2 Clinical setting

A lower AAD risk with probiotics was observed in both inpatient and outpatient settings. Among the 2 SRs/MAs that reported stratified results, the pooled RR was 0.48 (95% CI 0.25–0.94, *p* = 0.032) for inpatients, although heterogeneity was considerable (*I*^2^ = 94.6%, *p* < 0.001). For outpatients, the pooled RR was 0.63 (95% CI 0.48–0.82, *p* = 0.001) with no evidence of heterogeneity (*I*^2^ = 0.0%, *p* = 0.468). There was no clear difference between settings (*p* = 0.467), but only two reviews contributed and the inpatient estimate was highly heterogeneous, so this comparison is uninformative.

##### 3.5.2.3 Number of probiotic strains

Both multi-strain formulations and single-strain preparations were associated with a lower AAD incidence. The pooled RR was 0.57 (95% CI 0.48–0.69, *p* < 0.001) for multi-strain products and 0.55 (95% CI 0.46–0.67, *p* < 0.001) for single-strain products.

No difference between the two groups was detected (*p* = 0.791; four SRs/MAs contributed). This does not show that single-strain and multi-strain products are equivalent: each category pools many different strains and species, and a multi-strain product should be judged against its own single-strain components.

##### 3.5.2.4 Probiotic dose

A difference between dose categories was observed. This analysis rests on three SRs/MAs (18, 28, 31), two of which focus on children (18, 31) and share primary trials. Although lower doses (<5 × 10^9^ CFU/day) were associated with a lower AAD risk (RR 0.72, 95% CI 0.56–0.93, *p* = 0.011), higher doses (≥5 × 10^9^ CFU/day) were associated with a larger estimated risk reduction (RR 0.35, 95% CI 0.27–0.44, *p* < 0.001). The between-category difference had *p* < 0.001. With only two categories, three overlapping SRs/MAs, and age and product type varying between SRs/MAs, this finding is exploratory: it does not establish a dose–response relationship and cannot be transferred to individual products, some of which are used at recommended doses below the cut-off.

##### 3.5.2.5 Risk of bias

When stratified by risk-of-bias category, the pooled effect estimates remained consistently in favor of probiotics across all strata. Specifically, the pooled RR was 0.53 (95% CI 0.43–0.67, *p* < 0.001) in review-reported estimates classified as high risk of bias (*I*^2^ = 42.1%, *p* = 0.159), 0.62 (95% CI 0.53–0.73, *p* < 0.001) among those at low risk of bias (*I*^2^ = 26.0%, *p* = 0.240), 0.56 (95% CI 0.42–0.76, *p* < 0.001) for low/unclear risk of bias (*I*^2^ = 77.3%, *p* = 0.036), and 0.52 (95% CI 0.42–0.63, *p* < 0.001) in the unclear group (*I*^2^ = 0.0%, *p* = 0.404). No evidence of a difference between strata was found (*p* = 0.526), estimates did not vary materially by the risk-of-bias category reported in the source SRs/MAs (six SRs/MAs contributed). This does not address the low methodological quality of the SRs/MAs themselves.

#### 3.5.3 Sensitivity analysis and publication bias

Given the substantial heterogeneity in the primary RR analysis, a leave-one-out sensitivity analysis was performed. Exclusion of Zhang et al. (34) did not materially alter the pooled effect estimate (RR 0.54, 95% CI 0.50–0.58), but reduced heterogeneity substantially (*I*^2^ = 25.9%, *p* = 0.157). The pooled estimate was therefore unchanged, and the finding suggests that variation in the older adult population may have contributed disproportionately to between-SRs/MAs heterogeneity.

Further sensitivity analyses addressed methodological confidence and primary-study overlap are summarized in Supplementary Table S8 and Supplementary Figures S1–S3. Across all leave-one-review-out analyses the pooled RR ranged from 0.54 to 0.55. Restricting the analysis to the seven RR-reporting SRs/MAs rated high or low quality by AMSTAR 2 (20, 23, 24, 26–28, 31) gave RR 0.52 (95% CI 0.48–0.57; *I*^2^ = 3.4%). In subsets of SRs/MAs with low pairwise overlap the pooled estimates were weaker, less precise, and heterogeneous: RR 0.59 (95% CI 0.45–0.79; *I*^2^ = 87.8%; six SRs/MAs (21–23, 28, 34, 36), all pairwise CCA < 15%) and RR 0.63 (95% CI 0.47–0.85; *I*^2^ = 88.2%; five SRs/MAs (20, 22, 23, 34, 36), all pairwise CCA < 10%). These analyses indicate that the direction of the association did not depend on any single review, on the lower-quality reviews, or on the most overlapping reviews, but that its magnitude is smaller and its uncertainty larger when overlap is limited. They do not remove double counting of trials, and they do not show that the underlying evidence is robust.

Visual inspection of the funnel plot suggested asymmetry in the distribution of included SRs/MAs (Figure 6a). Egger’s test further indicated a potential small-study effect (*p* = 0.017), although the interpretation should be cautious due to heterogeneity among studies (Figure 6b). Both tests were applied to review-level estimates that share primary trials, so they cannot show whether individual trials were selectively unreported; that would require a funnel plot and asymmetry test on the 170 unique RCTs.

**Figure 6:**
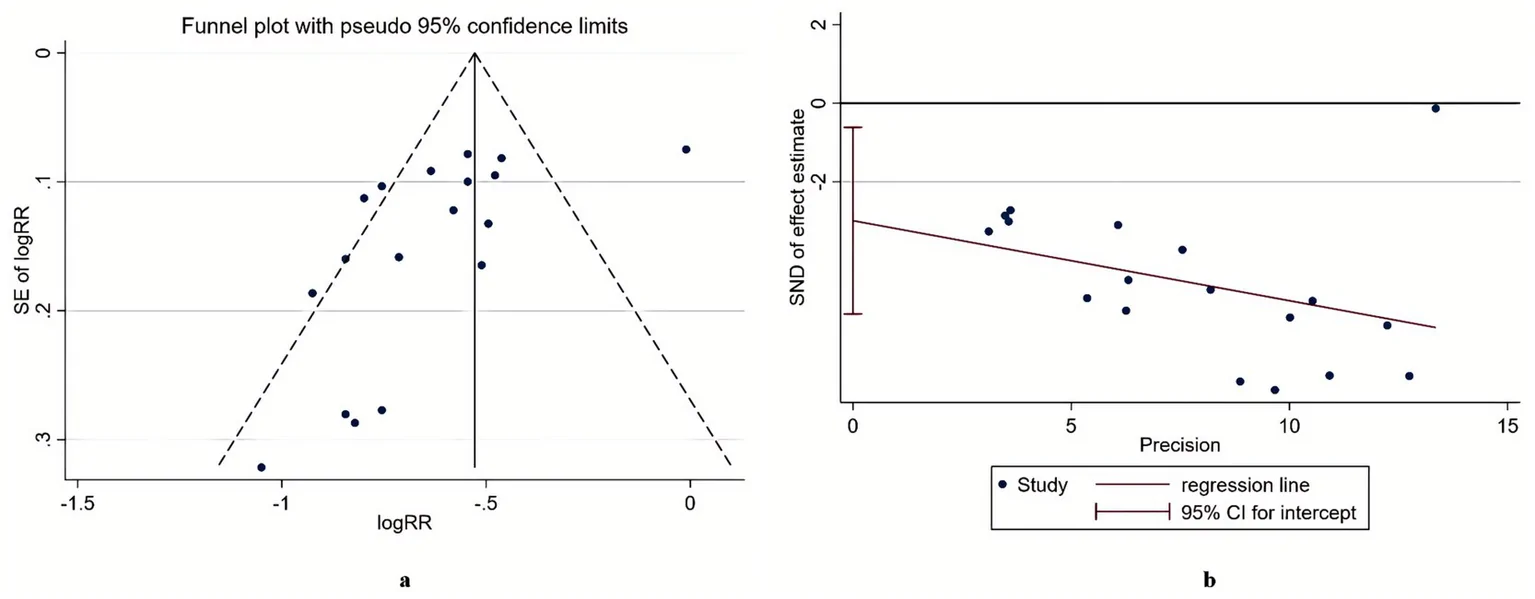
Assessment of publication bias. **(a)** Funnel plot. **(b)** Egger’s Regression test.

### 3.6 Safety assessment and adverse events

Four SRs/MAs reported adverse events between probiotics and placebo, but the efficacy indicators differed. Since these measures could not be combined separately, baseline event rates from the baseline trial had to be counted, so only the two SRs/MAs reporting RD were combined (28, 31). The random-effects pooled RD was 0.00 (95% CI −0.01 to 0.01), indicating no evidence of a difference in adverse event rates between the probiotic and placebo groups (Figure 7). Heterogeneity was low (*I*^2^ = 0.0%, *p* = 1.000), suggesting consistent findings across SRs/MAs. Thus, no evidence of increased adverse events was found, although only two SRs/MAs reported this outcome and adverse-event reporting was incomplete.

**Figure 7:**
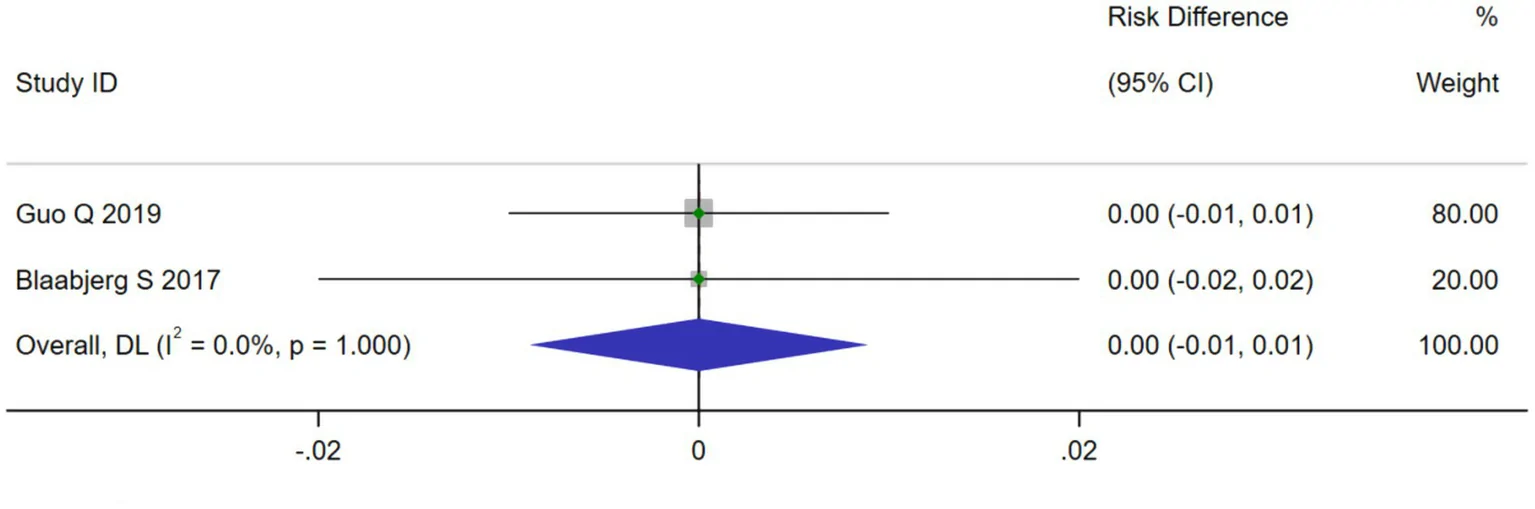
Forest plot of adverse events.

## 4 Discussion

This umbrella review synthesizes the review-level evidence currently available on probiotics for the prevention of AAD. Across 21 SRs/MAs covering 170 unique RCTs, probiotics were associated with a lower risk of AAD (RR 0.54, 95% CI 0.48–0.61), and this association was seen in most subgroup analyses. However, 20 of the 21 SRs/MAs were of low or critically low methodological quality, the SRs/MAs overlap considerably, and the interventions were heterogeneous; the evidence is therefore of low certainty. The absence of an excess of adverse events further supports the view that probiotics are generally well tolerated in this setting. Taken together, the findings are compatible with a beneficial effect of some probiotic products during antibiotic exposure, but they cannot identify which products, strains, or doses are effective, and the magnitude and certainty of benefit are not uniform across populations.

Biologically, the protective effect is coherent with the known consequences of antibiotic-induced dysbiosis. Antibiotics disrupt microbial diversity, diminish colonization resistance, and alter mucosal and immune homeostasis, creating conditions that favor opportunistic pathogen expansion and diarrhea. Probiotics may attenuate these processes through multiple, partly overlapping pathways, including competitive exclusion of pathogens, restoration of microbial function, reinforcement of epithelial barrier integrity, and modulation of host immune signaling (37). The consistency of benefit across both single-strain and multi-strain formulations suggests that efficacy is not determined solely by the number of organisms administered, but likely reflects a combination of strain identity, viability, dosage, and host context. This strain-and context-dependence is an important consideration, because treating probiotics as a uniform class may obscure clinically meaningful differences between formulations.

The absence of a difference between single-strain and multi-strain products should not be read as meaning that the choice makes no difference. Multi-strain products may be designed for different reasons: to combine strains with distinct resistance to the antibiotic in use, or to achieve greater efficacy by combining species. In the latter case a general pharmacological principle applies, namely that a combination is justified only if it has been shown to be better than its individual components. Our comparison of pooled categories across SRs/MAs cannot test this; head-to-head trials of a multi-strain product against its components would be needed.

A notable but tentative observation was that estimates differed by age group. Pooled estimates were similar in children and adults, whereas in older adults (older than 65 years) the pooled estimate was close to the null with a wide CI (RR 0.96, 95% CI 0.85–1.08). This stratum rests on four SRs/MAs, one of which contributed about two thirds of the weight (34), and the comparison is made across reviews with overlapping trials; we therefore regard it as a signal that needs confirmation. The finding is also consistent with the broader view that microbiome-targeted interventions are unlikely to be equally effective across all physiologic states (38–40). This may be attributed to reduced gut microbiota diversity and immunosenescence in the elderly, leading to increased colonization resistance against exogenous probiotics (41, 42). Although the evidence in older adults remains limited, the absence of a clear signal in this subgroup argues against extrapolating efficacy from younger populations without qualification. It is worth noting that recent studies suggest that early use of probiotics may to some extent prevent AAD in elderly patients, although this depends on the timing of intervention and the specificity of the strain.

Regarding clinical setting, AAD is more frequent in hospitalized patients than in outpatients, possibly because more severe antibiotic regimens are used in hospital. The pooled RR was numerically lower in inpatients (0.48) than in outpatients (0.63), which would be compatible with a larger benefit where the baseline risk is higher. However, only two reviews contributed, the inpatient estimate was highly heterogeneous (*I*^2^ = 94.6%), and the difference between settings was not clear (*p* = 0.467); no conclusion about setting can be drawn.

The difference between dose categories observed here is compatible with a dose-related effect, but it is exploratory: it rests on three overlapping SRs/MAs, two of them in children, compares only two categories, and is confounded by age and product type. At the same time, dose should not be interpreted in isolation: formulation stability, timing relative to antibiotic exposure, and strain-specific properties are all likely to influence outcomes. The current evidence base is not mature enough to define an optimal CFU threshold. The 5 × 10^9^ CFU/day cut-off is a pragmatic cut-point applied across heterogeneous products; it does not apply to every product (for instance, some products have recommended daily doses below this value) and should not be used for product selection in practice. Dose–response relationships should be examined in trials of specific products.

Methodological appraisal revealed substantial limitations in the evidence base. Most included SRs/MAs were rated low or critically low quality according to AMSTAR 2, largely because of absent protocols and incomplete reporting of excluded studies. These deficiencies mean that the pooled effect must be regarded as low-certainty evidence: only one SR/MA was rated high quality (23), and confidence in the results of the other 20 is limited. We did not apply GRADE, but the low to critically low quality of the SRs/MAs, their overlap, and the heterogeneity of interventions each argue for at most weak conclusions. Restricting the analysis to higher-quality SRs/MAs gave a similar estimate, but a similar estimate from overlapping SRs/MAs does not make the underlying trials more reliable. Moreover, the moderate overlap among SRs/MAs indicates that the literature has repeatedly synthesized a relatively small core of primary trials. In an umbrella review, such redundancy is not merely a bibliographic issue: it affects the apparent breadth of evidence and can create a false impression of independent replication. The present findings therefore reflect a large body of published work, but not necessarily a proportionally large body of independent data.

The pooled estimate was stable in sensitivity analyses, although this refers to statistical stability only and not to the robustness of the evidence, and heterogeneity remained an important feature of the dataset. Removal of the review focused on older adults substantially reduced between-review heterogeneity without materially changing the pooled estimate, reinforcing the view that population composition is a major source of variability. Heterogeneity in probiotic research is not simply statistical noise, but may represent real effect modification driven by age, disease context, antibiotic regimen, and probiotic formulation. Such variability also complicates the translation of pooled estimates into clinical recommendations, because a positive average effect can coexist with null benefit in clinically important subgroups.

Signals of small-study effects further temper the certainty of the evidence. Funnel plot asymmetry and Egger’s test raise the possibility that smaller studies with less favorable results are underrepresented, although these patterns may also arise from genuine heterogeneity or differences in study design. In a literature dominated by small trials and diverse probiotic preparations, publication bias is difficult to distinguish from clinical variation. Nevertheless, the presence of such signals reinforces the need for cautious interpretation and for future trials with prespecified protocols and complete outcome reporting. A funnel plot and asymmetry test on the 170 unique RCTs would be required to draw a firm conclusion about selective under-reporting at the trial level; in the present review these tests were applied to review-level estimates that share trials and are not a substitute.

Safety remains a central issue for any intervention intended for broad use during antibiotic therapy. In the present analysis, probiotics did not increase adverse events compared with placebo, which is reassuring and consistent with their generally favorable tolerability profile. However, adverse event reporting was limited, and rare harms are unlikely to be captured reliably in aggregate analyses. This is particularly relevant for severely immunocompromised or critically ill patients, in whom case reports of probiotic-associated bloodstream infections have been described. Accordingly, a favorable safety signal at the population level should not be interpreted as proof of universal safety across all clinical settings.

From a clinical perspective, the present findings are compatible with a preventive effect of some probiotic products against AAD in children and adults, but at low certainty. However, the evidence does not justify a blanket recommendation for all patient groups or all probiotic products. The effect appears to be contingent on age, dose, and probably strain-level characteristics, and these dependencies limit direct generalization. A more precise approach—one that aligns formulation choice with patient context and antibiotic exposure—will be more consistent with the evidence than a class-wide endorsement. In particular, these results should not be used to claim that any given probiotic product prevents AAD: such a claim requires product-specific evidence from adequately powered trials of that product.

Two features limit what this umbrella review can say about individual products. First, probiotic products differ in genus, species, strain, viable cell count, formulation, and manufacturing quality, and the actual content of commercial products may deviate from the label. Pooling across such products yields an average effect over a heterogeneous group of interventions, which is unlikely to apply to any particular product. Second, an umbrella review synthesizes published SRs/MAs, which themselves pooled trials of different interventions and populations; differences between interventions and patient groups that were already blurred in the source reviews cannot be recovered at the umbrella level, and subgroup comparisons across reviews are affected by overlapping trials. These limitations are intrinsic to the design. The pooled estimate should therefore be read as a summary of what published SRs/MAs report about probiotics as a broad category, not as evidence that any product is effective, and it should not be cited in support of marketing or clinical claims for specific products.

The main limitation of this umbrella review is that it inherits the constraints of the underlying SRs/MAs and RCTs. Differences in AAD definitions, antibiotic regimens, probiotic formulations, follow-up duration, and outcome ascertainment all contribute to heterogeneity and reduce comparability. In addition, the available evidence is skewed toward studies published in adults and children, with relatively sparse data in older adults and other high-risk populations. As a result, the conclusions are strongest for the general preventive effect of probiotics and weaker for identifying the most effective product, dose, or target population. Additional limitations are: (i) residual overlap, since 48.24% of the unique RCTs appear in two or more reviews and 71 of 210 review pairs show very high overlap, so the confidence intervals of the pooled estimates are probably too narrow and the sensitivity analyses cannot fully correct this; (ii) we did not re-analyze the 170 unique trials, so trial-level effects, heterogeneity, and publication bias could not be assessed; (iii) subgroup analyses used stratum-specific estimates reported by the reviews, several reviews contributed more than one estimate, and only two to four reviews contributed to the analyses of older adults, setting, strain number, and dose; (iv) certainty of evidence was not graded with GRADE; and (v) information on strain, formulation, and product quality was not available for evaluation.

Future research should move beyond the question of whether probiotics work in general and focus instead on which probiotic, at what dose, in whom, and under what antibiotic exposure. Large, rigorously designed trials with strain-specific interventions, standardized AAD definitions, adequate adverse-event surveillance, and age-stratified analyses are needed. Parallel efforts should improve the quality of systematic reviews through protocol registration, transparent reporting, and formal certainty-of-evidence assessment. Such work will be essential for transforming a broadly positive but heterogeneous evidence base into clinically actionable guidance.

## 5 Conclusion

Published SRs/MAs associate probiotics with a lower risk of antibiotic-associated diarrhea, without evidence of increased adverse events. However, the evidence is of low certainty: 13 of 21 SRs/MAs were of critically low methodological quality, the SRs/MAs share many primary trials, and the interventions were heterogeneous. These findings do not show that any specific probiotic product is effective. Well-designed, strain-specific RCTs and higher-quality SRs/MAs are needed to strengthen the evidence base, especially for older adults.

## Data Availability

The original contributions presented in the study are included in the article/Supplementary material, further inquiries can be directed to the corresponding author.
