# Supplementary material for: Probiotics for the prevention of antibiotic-associated diarrhea: an umbrella review and second-order meta-analysis of 21 systematic reviews covering 170 unique randomized controlled trials
Source: Front Nutr. 2026 Oct 27;13:1994645. doi: 10.3389/fnut.2026.1994645 (PMC13747751; doi:10.3389/fnut.2026.1994645)
Supplement: Supplementary file 1 [file Supplementary_file1.DOCX]

**Supplementary Table S1. PRIOR reporting checklist**

| Section topic | Item No | Checklist item | Location in manuscript | Status |
| --- | --- | --- | --- | --- |
| TITLE | | | | |
| Title | 1 | Identify the report as an overview of reviews. | Title | Reported |
| ABSTRACT |  |  |  |  |
| Abstract | 2 | Provide a comprehensive and accurate summary of the purpose, methods, and results of the overview of reviews. | Abstract | Reported |
| INTRODUCTION | | | | |
| Rationale | 3 | Describe the rationale for conducting the overview of reviews in the context of existing knowledge. | Introduction, paragraphs 1-5 | Reported |
| Objectives | 4 | Provide an explicit statement of the objective(s) or question(s) addressed by the overview of reviews. | Introduction, last paragraph | Reported |
| METHODS | | | | |
| Eligibility criteria | 5a | Specify the inclusion and exclusion criteria for the overview of reviews. If supplemental primary studies were included, this should be stated, with a rationale. | Section 2.3 | Reported |
|  | 5b | Specify the definition of 'systematic review' as used in the inclusion criteria for the overview of reviews. | Section 2.3 | Reported |
| Information sources | 6 | Specify all databases, registers, websites, organizations, reference lists, and other sources searched or consulted to identify systematic reviews and supplemental primary studies (if included). Specify the date when each source was last searched or consulted. | Section 2.2 | Reported |
| Search strategy | 7 | Present the full search strategies for all databases, registers and websites, such that they could be reproduced. Describe any search filters and limits applied. | Section 2.2; Supplementary Table S3 | Reported |
| Selection process | 8a | Describe the methods used to decide whether a systematic review or supplemental primary study (if included) met the inclusion criteria of the overview of reviews. | Section 2.3 | Reported |
|  | 8b | Describe how overlap in the populations, interventions, comparators, and/or outcomes of systematic reviews was identified and managed during study selection. | Sections 2.3 and 2.5 | Reported |
| Data collection process | 9a | Describe the methods used to collect data from reports. | Section 2.4 | Reported |
|  | 9b | If applicable, describe the methods used to identify and manage primary study overlap at the level of the comparison and outcome during data collection. For each outcome, specify the method used to illustrate and/or quantify the degree of primary study overlap across systematic reviews. | Section 2.5; Section 3.4; Figure 3; Supplementary Table S7 | Reported |
|  | 9c | If applicable, specify the methods used to manage discrepant data across systematic reviews during data collection. | Section 2.4; Section 4 | Not applicable |
| Data items | 10 | List and define all variables and outcomes for which data were sought. Describe any assumptions made and/or measures taken to identify and clarify missing or unclear information. | Sections 2.3 and 2.4 | Reported |
| Risk of bias assessment | 11a | Describe the methods used to assess risk of bias or methodological quality of the included systematic reviews. | Section 2.4 | Reported |
|  | 11b | Describe the methods used to collect data on (from the systematic reviews) and/or assess the risk of bias of the primary studies included in the systematic reviews. Provide a justification for instances where flawed, incomplete, or missing assessments are identified but not re-assessed. | Section 2.4; Section 3.2; Section 3.5.2.5 | Partly reported |
|  | 11c | Describe the methods used to assess the risk of bias of supplemental primary studies (if included). | - | Not applicable |
| Synthesis methods | 12a | Describe the methods used to summarize or synthesize results and provide a rationale for the choice(s). | Section 2.6 | Partly reported |
|  | 12b | Describe any methods used to explore possible causes of heterogeneity among results. | Section 2.6 | Reported |
|  | 12c | Describe any sensitivity analyses conducted to assess the robustness of the synthesized results. | Sections 2.5 and 2.6 | Reported |
| Reporting bias assessment | 13 | Describe the methods used to collect data on (from the systematic reviews) and/or assess the risk of bias due to missing results in a summary or synthesis (arising from reporting biases at the levels of the systematic reviews, primary studies, and supplemental primary studies, if included). | Section 2.6 | Reported |
| Certainty assessment | 14 | Describe the methods used to collect data on (from the systematic reviews) and/or assess certainty (or confidence) in the body of evidence for an outcome. | - | Not reported |
| RESULTS | | | | |
| Systematic review and supplemental primary study selection | 15a | Describe the results of the search and selection process, including the number of records screened, assessed for eligibility, and included in the overview of reviews, ideally with a flow diagram. | Section 3.1; Figure 1 | Reported |
|  | 15b | Provide a list of studies that might appear to meet the inclusion criteria, but were excluded, with the main reason for exclusion. | Section 3.1; Supplementary Table S5 | Reported |
| Characteristics of systematic reviews and supplemental primary studies | 16 | Cite each included systematic review and supplemental primary study (if included) and present its characteristics. | Section 3.2; Table 1 | Reported |
| Primary study overlap | 17 | Describe the extent of primary study overlap across the included systematic reviews. | Section 3.4; Figure 3; Supplementary Table S7 | Reported |
| Risk of bias in systematic reviews, primary studies, and supplemental primary studies | 18a | Present assessments of risk of bias or methodological quality for each included systematic review. | Section 3.3; Figure 2; Supplementary Table S6 | Reported |
|  | 18b | Present assessments (collected from systematic reviews or assessed anew) of the risk of bias of the primary studies included in the systematic reviews. | Section 3.2; Section 3.5.2.5; Figure 5e | Partly reported |
|  | 18c | Present assessments of the risk of bias of supplemental primary studies (if included). | - | Not applicable |
| Summary or synthesis of results | 19a | For all outcomes, summarize the evidence from the systematic reviews and supplemental primary studies (if included). If meta-analyses were done, present for each the summary estimate and its precision and measures of statistical heterogeneity. If comparing groups, describe the direction of the effect. | Sections 3.5.1 and 3.6; Figures 4 and 7 | Reported |
|  | 19b | If meta-analyses were done, present results of all investigations of possible causes of heterogeneity. | Section 3.5.2; Figure 5 | Reported |
|  | 19c | If meta-analyses were done, present results of all sensitivity analyses conducted to assess the robustness of synthesized results. | Section 3.5.3; Supplementary Table S8; Figures S1-S3 | Reported |
| Reporting biases | 20 | Present assessments (collected from systematic reviews and/or assessed anew) of the risk of bias due to missing primary studies, analyses, or results in a summary or synthesis (arising from reporting biases at the levels of the systematic reviews, primary studies, and supplemental primary studies, if included) for each summary or synthesis assessed. | Section 3.5.3; Figure 6 | Partly reported |
| Certainty of evidence | 21 | Present assessments (collected or assessed anew) of certainty (or confidence) in the body of evidence for each outcome. | - | Not reported |
| DISCUSSION | | | | |
| Discussion | 22a | Summarize the main findings, including any discrepancies in findings across the included systematic reviews and supplemental primary studies (if included). | Section 4, paragraph 1 and age paragraph | Reported |
|  | 22b | Provide a general interpretation of the results in the context of other evidence. | Section 4 | Reported |
|  | 22c | Discuss any limitations of the evidence from systematic reviews, their primary studies, and supplemental primary studies (if included) included in the overview of reviews. Discuss any limitations of the overview of reviews methods used. | Section 4 (methodological appraisal, small-study effects, limitations) | Reported |
|  | 22d | Discuss implications for practice, policy, and future research (both systematic reviews and primary research). Consider the relevance of the findings to the end users of the overview of reviews, e.g., healthcare providers, policymakers, patients, among others. | Section 4 (clinical perspective, future research); Section 5 | Reported |
| OTHER INFORMATION |  |  |  |  |
| Registration and protocol | 23a | Provide registration information for the overview of reviews, including register name and registration number, or state that the overview of reviews was not registered. | Abstract; Section 2.1 | Reported |
|  | 23b | Indicate where the overview of reviews protocol can be accessed, or state that a protocol was not prepared. | Section 2.1 | Partly reported |
|  | 23c | Describe and explain any amendments to information provided at registration or in the protocol. Indicate the stage of the overview of reviews at which amendments were made. | Section 2.5 | Reported |
| Support | 24 | Describe sources of financial or non-financial support for the overview of reviews, and the role of the funders or sponsors in the overview of reviews. | Acknowledgements; Funding | Partly reported |
| Competing interests | 25 | Declare any competing interests of the overview of reviews' authors. | Conflict of Interest | Reported |
| Author information | 26a | Provide contact information for the corresponding author. | Title page | Reported |
|  | 26b | Describe the contributions of individual authors and identify the guarantor of the overview of reviews. | Authorship contribution | Reported |
| Availability of data and other materials | 27 | Report which of the following are available, where they can be found, and under which conditions they may be accessed: template data collection forms; data collected from included systematic reviews and supplemental primary studies; analytic code; any other materials used in the overview of reviews. | Data Availability Statement | Reported |

**Reference:** Gates M, Gates A, Pieper D, et al. Reporting guideline for overviews of reviews of healthcare interventions: development of the PRIOR statement. BMJ 2022;378:e070849.

**Supplementary Table S2. PRISMA 2020 reporting checklist**

| Section and topic | Item No | Checklist item | Location in manuscript | Status |
| --- | --- | --- | --- | --- |
| TITLE | | | | |
| Title | 1 | Identify the report as a systematic review. | Title | Reported |
| ABSTRACT | | | | |
| Abstract | 2 | See the PRISMA 2020 for Abstracts checklist. | Abstract; Table S2b | Partly reported |
| INTRODUCTION | | | | |
| Rationale | 3 | Describe the rationale for the review in the context of existing knowledge. | Introduction | Reported |
| Objectives | 4 | Provide an explicit statement of the objective(s) or question(s) the review addresses. | Introduction, last paragraph | Reported |
| METHODS | | | | |
| Eligibility criteria | 5 | Specify the inclusion and exclusion criteria for the review and how studies were grouped for the syntheses. | Sections 2.3 and 2.6 | Reported |
| Information sources | 6 | Specify all databases, registers, websites, organisations, reference lists and other sources searched or consulted to identify studies. Specify the date when each source was last searched or consulted. | Section 2.2 | Reported |
| Search strategy | 7 | Present the full search strategies for all databases, registers and websites, including any filters and limits used. | Section 2.2; Supplementary Table S3 | Reported |
| Selection process | 8 | Specify the methods used to decide whether a study met the inclusion criteria of the review, including how many reviewers screened each record and each report retrieved, whether they worked independently, and if applicable, details of automation tools used in the process. | Section 2.3 | Reported |
| Data collection process | 9 | Specify the methods used to collect data from reports, including how many reviewers collected data from each report, whether they worked independently, any processes for obtaining or confirming data from study investigators, and if applicable, details of automation tools used in the process. | Sections 2.3 and 2.4 | Reported |
| Data items | 10a | List and define all outcomes for which data were sought. Specify whether all results that were compatible with each outcome domain in each study were sought (e.g. for all measures, time points, analyses), and if not, the methods used to decide which results to collect. | Sections 2.3 and 2.4 | Partly reported |
|  | 10b | List and define all other variables for which data were sought (e.g. participant and intervention characteristics, funding sources). Describe any assumptions made about any missing or unclear information. | Section 2.4; Section 2.3 | Reported |
| Study risk of bias assessment | 11 | Specify the methods used to assess risk of bias in the included studies, including details of the tool(s) used, how many reviewers assessed each study and whether they worked independently, and if applicable, details of automation tools used in the process. | Section 2.4 | Reported |
| Effect measures | 12 | Specify for each outcome the effect measure(s) (e.g. risk ratio, mean difference) used in the synthesis or presentation of results. | Section 2.6 | Reported |
| Synthesis methods | 13a | Describe the processes used to decide which studies were eligible for each synthesis (e.g. tabulating the study intervention characteristics and comparing against the planned groups for each synthesis (item #5)). | Sections 2.5 and 2.6 | Reported |
|  | 13b | Describe any methods required to prepare the data for presentation or synthesis, such as handling of missing summary statistics, or data conversions. | Section 2.6 | Reported |
|  | 13c | Describe any methods used to tabulate or visually display results of individual studies and syntheses. | Table 1; Figures 2-7 | Partly reported |
|  | 13d | Describe any methods used to synthesize results and provide a rationale for the choice(s). If meta-analysis was performed, describe the model(s), method(s) to identify the presence and extent of statistical heterogeneity, and software package(s) used. | Section 2.6 | Partly reported |
|  | 13e | Describe any methods used to explore possible causes of heterogeneity among study results (e.g. subgroup analysis, meta-regression). | Section 2.6 | Reported |
|  | 13f | Describe any sensitivity analyses conducted to assess robustness of the synthesized results. | Sections 2.5 and 2.6 | Reported |
| Reporting bias assessment | 14 | Describe any methods used to assess risk of bias due to missing results in a synthesis (arising from reporting biases). | Section 2.6 | Reported |
| Certainty assessment | 15 | Describe any methods used to assess certainty (or confidence) in the body of evidence for an outcome. | - | Not reported |
| RESULTS | | | | |
| Study selection | 16a | Describe the results of the search and selection process, from the number of records identified in the search to the number of studies included in the review, ideally using a flow diagram. | Section 3.1; Figure 1 | Reported |
|  | 16b | Cite studies that might appear to meet the inclusion criteria, but which were excluded, and explain why they were excluded. | Section 3.1; Supplementary Table S5 | Reported |
| Study characteristics | 17 | Cite each included study and present its characteristics. | Section 3.2; Table 1 | Reported |
| Risk of bias in studies | 18 | Present assessments of risk of bias for each included study. | Section 3.3; Figure 2; Supplementary Table S6 | Reported |
| Results of individual studies | 19 | For all outcomes, present, for each study: (a) summary statistics for each group (where appropriate) and (b) an effect estimate and its precision (e.g. confidence/credible interval), ideally using structured tables or plots. | Figures 4 and 7; Supplementary Table S4 | Reported |
| Results of syntheses | 20a | For each synthesis, briefly summarise the characteristics and risk of bias among contributing studies. | Sections 3.2, 3.3 and 3.5 | Partly reported |
|  | 20b | Present results of all statistical syntheses conducted. If meta-analysis was done, present for each the summary estimate and its precision (e.g. confidence/credible interval) and measures of statistical heterogeneity. If comparing groups, describe the direction of the effect. | Sections 3.5.1 and 3.6 | Reported |
|  | 20c | Present results of all investigations of possible causes of heterogeneity among study results. | Section 3.5.2; Figure 5 | Reported |
|  | 20d | Present results of all sensitivity analyses conducted to assess the robustness of the synthesized results. | Section 3.5.3; Supplementary Table S8; Figures S1-S3 | Reported |
| Reporting biases | 21 | Present assessments of risk of bias due to missing results (arising from reporting biases) for each synthesis assessed. | Section 3.5.3; Figure 6 | Partly reported |
| Certainty of evidence | 22 | Present assessments of certainty (or confidence) in the body of evidence for each outcome assessed. | - | Not reported |
| DISCUSSION | | | | |
| Discussion | 23a | Provide a general interpretation of the results in the context of other evidence. | Section 4 | Reported |
|  | 23b | Discuss any limitations of the evidence included in the review. | Section 4 | Reported |
|  | 23c | Discuss any limitations of the review processes used. | Section 4, limitations paragraph | Reported |
|  | 23d | Discuss implications of the results for practice, policy, and future research. | Section 4; Section 5 | Reported |
| OTHER INFORMATION | | | | |
| Registration and protocol | 24a | Provide registration information for the review, including register name and registration number, or state that the review was not registered. | Abstract; Section 2.1 | Reported |
|  | 24b | Indicate where the review protocol can be accessed, or state that a protocol was not prepared. | Section 2.1 | Partly reported |
|  | 24c | Describe and explain any amendments to information provided at registration or in the protocol. | Section 2.5 | Reported |
| Support | 25 | Describe sources of financial or non-financial support for the review, and the role of the funders or sponsors in the review. | Acknowledgements; Funding | Partly reported |
| Competing interests | 26 | Declare any competing interests of review authors. | Conflict of Interest | Reported |
| Availability of data, code and other materials | 27 | Report which of the following are publicly available and where they can be found: template data collection forms; data extracted from included studies; data used for all analyses; analytic code; any other materials used in the review. | Data Availability Statement | Partly reported |

**Reference:** Page MJ, McKenzie JE, Bossuyt PM, et al. The PRISMA 2020 statement: an updated guideline for reporting systematic reviews. BMJ 2021;372:n71.

**Supplementary Table S3. Database-specific search strategies**

| Database |  | Search strategies |
| --- | --- | --- |
| PubMed | #1 | "Prebiotics"[Mesh] OR "Probiotics"[Mesh] OR "Synbiotics"[Mesh] OR Probiotic*[Title/Abstract] OR Prebiotic*[Title/Abstract] OR Synbiotic*[Title/Abstract] OR bifidobacteri*[Title/Abstract] OR lactobacill*[Title/Abstract] OR Streptococc*[Title/Abstract] OR Saccharomyces[Title/Abstract] OR bacill*[Title/Abstract] |
|  | #2 | "Diarrhea"[Mesh] OR Diarrhea*[Title/Abstract] OR Diarrhoea*[Title/Abstract] |
|  | #3 | "Meta-Analysis" [Publication Type] OR "Meta-Analysis as Topic"[Mesh] OR "Systematic Review" [Publication Type] OR "Systematic Reviews as Topic"[Mesh] OR meta analysis[Title/Abstract] OR meta analyses[Title/Abstract] OR metaanalysis[Title/Abstract] OR metanalysis[Title/Abstract] OR met-analysis[Title/Abstract] OR metaanalyses[Title/Abstract] OR metanalyses[Title/Abstract] OR met-analyses[Title/Abstract] OR systematic review[Title/Abstract] OR systematic reviews[Title/Abstract] |
|  | #4 | #1 AND #2 AND #3 |
| Embase | #1 | ('prebiotic agent'/exp OR 'probiotic agent'/exp OR probiotic*:ti,ab,kw OR prebiotic*:ti,ab,kw OR synbiotic*:ti,ab,kw OR bifidobacteri*:ti,ab,kw OR lactobacill*:ti,ab,kw OR streptococc*:ti,ab,kw OR saccharomyces:ti,ab,kw OR bacill*:ti,ab,kw) |
|  | #2 | ('diarrhea'/exp OR diarrhea*:ti,ab,kw OR diarrhoea*:ti,ab,kw) |
|  | #3 | ('meta analysis'/exp OR 'meta analysis(topic)'/exp OR 'systematic review'/exp OR 'systematic review(topic)'/exp OR 'meta analysis':ab,ti,kw OR 'meta analyses':ab,ti,kw OR 'metaanalysis':ab,ti,kw OR 'metanalysis':ab,ti,kw OR 'met-analysis':ab,ti,kw OR 'metaanalyses':ab,ti,kw OR 'metanalyses':ab,ti,kw OR 'met-analyses':ab,ti,kw OR 'systematic review':ab,ti,kw OR 'systematic reviews':ab,ti,kw) |
|  | #4 | #1 AND #2 AND #3 |
| Web of Science | #1 | TS=(Diarrhea* OR Diarrhoea*) |
|  | #2 | TS=(Probiotic* OR Prebiotic* OR Synbiotic* OR bifidobacteri* OR lactobacill* OR Streptococc* OR Saccharomyces OR bacill*) |
|  | #3 | TS=('meta analysis' OR 'meta analyses' OR 'metaanalysis' OR 'metanalysis' or 'met-analysis' OR 'metaanalyses' or 'metanalyses' OR 'met-analyses' OR 'systematic review' OR 'systematic reviews') |
|  | #4 | #1 AND #2 AND #3 |
| CENTRAL | #1 | (Probiotic* or Prebiotic* or Synbiotic* or bifidobacteri* or lactobacill* or Streptococc* or Saccharomyces or bacill*).mp. [mp=title, original title, abstract, floating sub-heading word, mesh headings, heading words, keyword] |
|  | #2 | (Probiotic* or Prebiotic* or Synbiotic* or bifidobacteri* or lactobacill* or Streptococc* or Saccharomyces or bacill*).m_titl. |
|  | #3 | (Diarrhea* or Diarrhoea*).mp. [mp=title, original title, abstract, floating sub-heading word, mesh headings, heading words, keyword] |
|  | #4 | (Diarrhea* or Diarrhoea*).m_titl. |
|  | #5 | ("meta analysis" or "meta analyses" or "metaanalysis" or "metanalysis" or "met-analysis" or "metaanalyses" or "metanalyses" or "met-analyses" or "systematic review" or "systematic reviews" or "data pooling" or "data poolings" or "clinical trial overviews" or "clinical trial overview").mp. [mp=title, original title, abstract, floating sub-heading word, mesh headings, heading words, keyword] |
|  | #6 | ("meta analysis" or "meta analyses" or "metaanalysis" or "metanalysis" or "met-analysis" or "metaanalyses" or "metanalyses" or "met-analyses" or "systematic review" or "systematic reviews" or "data pooling" or "data poolings" or "clinical trial overviews" or "clinical trial overview").m_titl. |
|  | #7 | #1 OR #2 |
|  | #8 | #3 OR #4 |
|  | #9 | #5 OR #6 |
|  | #10 | #7 AND #8 AND #9 |
| CINAHL | #1 | SU (Probiotic* or Prebiotic* or Synbiotic* or bifidobacteri* or lactobacill* or Streptococc* or Saccharomyces or bacill*) |
|  | #2 | SU (Diarrhea* OR Diarrhoea*) |
|  | #3 | SU ("meta analysis" OR "meta analyses" OR "metaanalysis" OR "metanalysis" OR "met-analysis" OR "metaanalyses" OR "metanalyses" OR "met-analyses" OR "systematic review" OR "systematic reviews") |
|  | #4 | #1 AND #2 AND #3 |
| Cochrane Database of Systematic Reviews | #1 | (Probiotic* or Prebiotic* or Synbiotic* or bifidobacteri* or lactobacill* or Streptococc* or Saccharomyces or bacill*).mp. [mp=title, short title, abstract, full text, keywords, caption text] |
|  | #2 | (Probiotic* or Prebiotic* or Synbiotic* or bifidobacteri* or lactobacill* or Streptococc* or Saccharomyces or bacill*).m_titl. |
|  | #3 | (Diarrhea* or Diarrhoea*).mp. [mp=title, short title, abstract, full text, keywords, caption text] |
|  | #4 | (Diarrhea* or Diarrhoea*).m_titl. |
|  | #5 | ("meta analysis" or "meta analyses" or "metaanalysis" or "metanalysis" or "met-analysis" or "metaanalyses" or "metanalyses" or "met-analyses" or "systematic review" or "systematic reviews").mp. [mp=title, short title, abstract, full text, keywords, caption text] |
|  | #6 | ("meta analysis" or "meta analyses" or "metaanalysis" or "metanalysis" or "met-analysis" or "metaanalyses" or "metanalyses" or "met-analyses" or "systematic review" or "systematic reviews").m_titl. |
|  | #7 | #1 OR #2 |
|  | #8 | #3 OR #4 |
|  | #9 | #5 OR #6 |
|  | #10 | #7 AND #8 AND #9 |
| Scoups | #1 | ( TITLE-ABS-KEY ( Probiotic* OR Prebiotic* OR Synbiotic* OR bifidobacteri* OR lactobacill* OR Streptococc* OR Saccharomyces OR bacill* ) |
|  | #2 | TITLE-ABS-KEY ( Diarrhea* OR Diarrhoea* ) |
|  | #3 | TITLE-ABS-KEY ( "meta analysis" OR "meta analyses" OR "metaanalysis" OR "metanalysis" OR "met-analysis" OR "metaanalyses" OR "metanalyses" OR "met-analyses" OR "systematic review" OR "systematic reviews") |
|  | #4 | #1 AND #2 AND #3 |
| Cochrane library | #1 | MeSH descriptor: [Prebiotics] explode all trees |
|  | #2 | MeSH descriptor: [Probiotics] explode all trees |
|  | #3 | (Probiotic* OR Prebiotic* OR Synbiotic* OR bifidobacteri* OR lactobacill* OR Streptococc* OR Saccharomyces OR bacill*):ti,ab,kw |
|  | #4 | MeSH descriptor: [Diarrhea] explode all trees |
|  | #5 | (Diarrhea* OR Diarrhoea*):ti,ab,kw |
|  | #6 | MeSH descriptor: [Meta-Analysis] explode all trees |
|  | #7 | MeSH descriptor: [Meta-Analysis as Topic] explode all trees |
|  | #8 | MeSH descriptor: [Systematic Review] explode all trees |
|  | #9 | MeSH descriptor: [Systematic Reviews as Topic] explode all trees |
|  | #10 | ("meta analysis" or "meta analyses" or "metaanalysis" or "metanalysis" or "met-analysis" or "metaanalyses" or "metanalyses" or "met-analyses" or "systematic review" or "systematic reviews"):ti,ab,kw |
|  | #11 | #1 OR #2 OR #3 |
|  | #12 | #4 OR #5 |
|  | #13 | #6 OR #7 OR #8 OR #9 OR #10 |
|  | #14 | #11 AND #12 AND #13 |

**Note:** Searches covered each database from inception through March 21, 2026. Search strategies are presented using the database-specific syntax in which they were run.

**Abbreviations:** CENTRAL, Cochrane Central Register of Controlled Trials; CINAHL, Cumulative Index to Nursing and Allied Health Literature; MeSH, Medical Subject Headings.

**Supplementary Table S4a. Review-level effect estimates extracted from the included SRs/MAs**

| **Study ID** | **Outcome** | **Effect measure** | **Effect estimate** | **95% CI** | **I² (%)** | **Location in source review** |
| --- | --- | --- | --- | --- | --- | --- |
| Cremonini F 2002 | Incidence of AAD | RR | 0.3966 | 0.275-0.571 | NR | Page 3 (Results) |
| D'Souza AL 2002 | Incidence of AAD | OR | 0.37 | 0.26-0.52 | NR | Fig 5 |
| Johnston BC 2006 | Incidence of AAD | RR | 0.43 | 0.25-0.75 | 70.1 | Page 4 (Fig 2) |
| McFarland LV 2006 | Incidence of AAD | RR | 0.43 | 0.31-0.58 | NR | Page 7 (Figure 2) |
| Szajewska H 2006 | Incidence of AAD | RR | 0.44 | 0.25-0.77 | 69.6 | Page 4 (Figure) |
| Kale-Pradhan PB 2010 | Incidence of AAD | RR | 0.35 | 0.19-0.67 | 79 | Page 5 (Figure 2) |
| Avadhani A 2011 | Incidence of AAD | RR | 0.56 | 0.44-0.71 | NR | Page 5 (Table 3) |
| Hempel S 2012 | Incidence of AAD | RR | 0.58 | 0.50-0.68 | 54 | Page 1 (Results); page 8 (Figure) |
| Videlock EJ 2012 | Incidence of AAD | RR | 0.53 | 0.44-0.63 | 44 | Page 9 (Figure 2) |
| Pattani R 2013 | Incidence of AAD | RR | 0.61 | 0.47-0.79 | 39 | Page 6 (Figure 2A) |
| Szajewska H 2015 | Incidence of AAD | RR | 0.47 | 0.38-0.57 | 30 | Page 4 (Fig 1) |
| Jafarnejad S 2016 | Incidence of AAD | RR | 0.58 | 0.48-0.71 | 58 | Page 6 (Figure 2) |
| Blaabjerg S 2017 | Incidence of AAD | RR | 0.49 | 0.36-0.66 | 58 | Page 7 (Main Outcome) |
| Blaabjerg S 2017 | Adverse events | RD | 0.00 | -0.02-0.02 | 66 | Page 10 (Figure 5) |
| Xu HB 2017 | Incidence of AAD | OR | 0.33 | 0.29-0.39 | NR | Page 5 (Results) |
| Cai J 2018 | Incidence of AAD | OR | 0.44 | 0.36-0.54 | NR | Supplementary Material 4, Figure S4.1; Supplementary Material 5, Table S5.4 |
| Cai J 2018 | Adverse events | OR | 0.51 | 0.38-0.68 | NR | Supplementary Material 5, Figure S5.1 and Table S5.4 |
| Guo Q 2019 | Incidence of AAD | RR | 0.45 | 0.36-0.56 | 57 | Page 4 (Summary of findings) |
| Guo Q 2019 | Adverse events | RD | 0.00 | -0.01-0.01 | 75 | Page 4 (Summary of findings) |
| Goodman C 2021 | Incidence of AAD | RR | 0.63 | 0.54-0.73 | 60 | Page 9 (Figure 3) |
| Liao W 2021 | Incidence of AAD | RR | 0.62 | 0.51-0.74 | 58 | Page 8 (Figure 4) |
| Liao W 2021 | Adverse events | RR | 1 | 0.87-1.14 | 0 | Page 9 (Figure 5) |
| Zhang L 2022 | Incidence of AAD | RR | 0.99 | 0.85-1.14 | 49 | Page 5 (Figure 3) |
| Fadin L 2023 | Incidence of AAD | RR | 0.47 | 0.27-0.80 | 68 | Page 10 (Figure 2A) |
| Wanyama H 2025 | Incidence of AAD | RR | 0.6 | 0.43-0.82 | 78 | Page 7 (Figure 3) |

**Note:** Effect estimates were extracted at the review level. *I²* values are those reported in the source SRs/MAs; NR indicates that heterogeneity was not reported. “Location in source review” identifies where each estimate was obtained in the original publication.

**Abbreviations:** AAD, antibiotic-associated diarrhea; CI, confidence interval; I², I-squared statistic; NR, not reported; OR, odds ratio; RD, risk difference; RR, risk ratio; SR/MA, systematic review/meta-analysis.

**Supplementary Table S4b. Subgroup-specific effect estimates for AAD incidence extracted from the included SRs/MAs**

| **Study ID** | **No. of RCTs** | **RR** | **95% CI** | **I² (%)** | **Subgroup category** | **Subgroup domain** |
| --- | --- | --- | --- | --- | --- | --- |
| Kale-Pradhan PB 2010 | 6 | 0.24 | 0.08-0.75 | 83 | Adults | Age group |
| Hempel S 2012 | 14 | 0.54 | 0.34-0.85 | 45 | Adults | Age group |
| Videlock EJ 2012 | 24 | 0.53 | 0.43-0.66 | 44 | Adults | Age group |
| Szajewska H 2015 | 15 | 0.49 | 0.38-0.63 | NR | Adults | Age group |
| Jafarnejad S 2016 | 25 | 0.47 | 0.4-0.56 | 43 | Adults | Age group |
| Blaabjerg S 2017 | 9 | 0.53 | 0.37-0.76 | 47 | Adults | Age group |
| Wanyama H 2025 | 11 | 0.64 | 0.48-0.85 | 40 | Adults | Age group |
| Szajewska H 2006 | 6 | 0.44 | 0.25-0.77 | 69.6 | Adults | Age group |
| Avadhani A 2011 | 8 | 0.56 | 0.44-0.71 | NR | Adults | Age group |
| Pattani R 2013 | 15 | 0.61 | 0.47-0.79 | 39 | Adults | Age group |
| Jafarnejad S 2016 | 30 | 0.58 | 0.48-0.71 | 58 | Adults | Age group |
| Liao W 2021 | 36 | 0.62 | 0.51-0.74 | 58 | Adults | Age group |
| Wanyama H 2025 | 15 | 0.6 | 0.43-0.82 | 78 | Adults | Age group |
| Kale-Pradhan PB 2010 | 4 | 0.44 | 0.18-1.08 | 76 | Children | Age group |
| Hempel S 2012 | 16 | 0.55 | 0.38-0.80 | 68 | Children | Age group |
| Videlock EJ 2012 | 10 | 0.48 | 0.35-0.65 | 36 | Children | Age group |
| Szajewska H 2015 | 6 | 0.43 | 0.30-0.60 | NR | Children | Age group |
| Blaabjerg S 2017 | 8 | 0.42 | 0.23-0.77 | 76 | Children | Age group |
| Johnston BC 2006 | 6 | 0.43 | 0.25-0.75 | 70.1 | Children | Age group |
| Guo Q 2019 | 33 | 0.45 | 0.36-0.56 | 57 | Children | Age group |
| Fadin L 2023 | 12 | 0.47 | 0.27-0.80 | 68 | Children | Age group |
| Hempel S 2012 | 3 | 0.81 | 0.40-1.63 | 65 | Older adults | Age group |
| Jafarnejad S 2016 | 5 | 0.94 | 0.76-1.15 | 65 | Older adults | Age group |
| Wanyama H 2025 | 4 | 0.47 | 0.17-1.30 | 93 | Older adults | Age group |
| Zhang L 2022 | 8 | 0.99 | 0.85-1.14 | 49 | Older adults | Age group |
| McFarland LV 2006 | 7 | 0.51 | 0.38-0.68 | NR | Mixture | Number of probiotic strains |
| Guo Q 2019 | 13 | 0.53 | 0.37-0.75 | 39 | Mixture | Number of probiotic strains |
| Liao W 2021 | 28 | 0.58 | 0.45-0.75 | 64 | Mixture | Number of probiotic strains |
| Liao W 2021 | 20 | 0.61 | 0.49-0.76 | 60 | Mixture | Number of probiotic strains |
| Liao W 2021 | 12 | 0.41 | 0.27-0.63 | 71 | Mixture | Number of probiotic strains |
| Wanyama H 2025 | 3 | 0.90 | 0.68-1.20 | 33 | Mixture | Number of probiotic strains |
| Wanyama H 2025 | 5 | 0.40 | 0.21-0.76 | 62 | Mixture | Number of probiotic strains |
| McFarland LV 2006 | 6 | 0.46 | 0.21-1.03 | NR | Single | Number of probiotic strains |
| Guo Q 2019 | 20 | 0.42 | 0.32-0.56 | 62 | Single | Number of probiotic strains |
| Liao W 2021 | 8 | 0.62 | 0.52-0.75 | 0 | Single | Number of probiotic strains |
| Liao W 2021 | 15 | 0.64 | 0.44-0.93 | 56 | Single | Number of probiotic strains |
| Wanyama H 2025 | 7 | 0.60 | 0.36-1.01 | 84 | Single | Number of probiotic strains |
| Liao W 2021 | 4 | 0.69 | 0.51-0.92 | 0 | Community | Clinical setting |
| Liao W 2021 | 16 | 0.75 | 0.60-0.94 | 61 | Hospital | Clinical setting |
| Jafarnejad S 2016 | 22 | 0.67 | 0.58-0.77 | 64 | Inpatient | Clinical setting |
| Guo Q 2019 | 10 | 0.34 | 0.26-0.45 | 3 | Inpatient | Clinical setting |
| Jafarnejad S 2016 | 5 | 0.67 | 0.49-0.92 | 0 | Outpatient | Clinical setting |
| Guo Q 2019 | 11 | 0.54 | 0.33-0.88 | 75 | Outpatient | Clinical setting |
| Liao W 2021 | 12 | 0.49 | 0.33-0.72 | 43 | <10^10^ | Probiotic dose |
| Johnston BC 2006 | 1 | 0.96 | 0.61-1.50 | NR | <5×10⁹ CFU/d | Probiotic dose |
| Blaabjerg S 2017 | 6 | 0.61 | 0.42-0.90 | 40 | <5×10^9^ CFU/d | Probiotic dose |
| Guo Q 2019 | 12 | 0.68 | 0.46-1.01 | 53 | <5×10^9^ CFU/d | Probiotic dose |
| Liao W 2021 | 14 | 0.77 | 0.60-0.98 | 52 | ≥10^10^ | Probiotic dose |
| Johnston BC 2006 | 4 | 0.36 | 0.25-0.53 | 3.5 | ≥5×10⁹ CFU/d | Probiotic dose |
| Blaabjerg S 2017 | 4 | 0.18 | 0.08-0.42 | 41 | ≥5×10^9^ CFU/d | Probiotic dose |
| Guo Q 2019 | 20 | 0.37 | 0.30-0.46 | 36 | ≥5×10^9^ CFU/d | Probiotic dose |
| Goodman C 2021 | 4 | 0.54 | 0.38-0.76 | 0 | Higher dose | Probiotic dose |
| Videlock EJ 2012 | 24 | 0.56 | 0.45-0.69 | 50 | Any lactobacilli | Probiotic type |
| Fadin L 2023 | 5 | 0.59 | 0.21-1.71 | 78 | Association of species of probiotics | Probiotic type |
| Guo Q 2019 | 1 | 0.43 | 0.11-1.62 | NR | Bacillus clausii | Probiotic type |
| Goodman C 2021 | 1 | 0.61 | 0.41-0.89 | NR | Bacillus clausii | Probiotic type |
| Videlock EJ 2012 | 10 | 0.55 | 0.37-0.82 | 48 | Bifidobacteria | Probiotic type |
| Jafarnejad S 2016 | 13 | 0.77 | 0.65-0.92 | 63 | Bifidobacteria | Probiotic type |
| Goodman C 2021 | 6 | 0.7 | 0.54-0.91 | 19 | Bifidobacterium animalis ssp. Lactis | Probiotic type |
| Goodman C 2021 | 4 | 0.9 | 0.62-1.32 | 47 | Bifidobacterium bifidum | Probiotic type |
| Goodman C 2021 | 1 | 0.39 | 0.19-0.79 | NR | Bifidobacterium licheniformis | Probiotic type |
| Goodman C 2021 | 4 | 0.46 | 0.28-0.73 | 25 | Bifidobacterium longum | Probiotic type |
| Goodman C 2021 | 1 | 0.35 | 0.19-0.62 | NR | Bifidobacterium subtilis | Probiotic type |
| Szajewska H 2006 | 1 | 0.52 | 0.29-0.95 | NR | Bifidobacterium. lactis+Streptococcus. thermophilus | Probiotic type |
| Guo Q 2019 | 1 | 0.52 | 0.29-0.95 | NR | Bifidobacterium. lactis+Streptococcus. thermophilus | Probiotic type |
| Hempel S 2012 | 25 | 0.66 | 0.49-0.88 | NR | Blend | Probiotic type |
| Goodman C 2021 | 2 | 0.29 | 0.06-1.34 | 0 | Clostridium butyricum | Probiotic type |
| Hempel S 2012 | 3 | 0.51 | 0.38-0.68 | 0 | Enterococcus [Streptococcus ] faecium SF68 | Probiotic type |
| Hempel S 2012 | 17 | 0.64 | 0.47-0.86 | 56 | Lactobacillus | Probiotic type |
| Jafarnejad S 2016 | 22 | 0.66 | 0.57-0.77 | 67 | Lactobacillus | Probiotic type |
| Goodman C 2021 | 28 | 0.63 | 0.2-0.76 | 61 | Lactobacillus | Probiotic type |
| Liao W 2021 | 12 | 0.67 | 0.50-0.91 | 44 | Lactobacillus | Probiotic type |
| Fadin L 2023 | 12 | 0.47 | 0.27-0.80 | 68 | Lactobacillus | Probiotic type |
| Kale-Pradhan PB 2010 | 10 | 0.35 | 0.19-0.67 | 79 | Lactobacillus | Probiotic type |
| Goodman C 2021 | 18 | 0.66 | 0.54-0.80 | 50 | Lactobacillus acidophilus | Probiotic type |
| Blaabjerg S 2017 | 2 | 0.79 | 0.47-1.33 | 0 | Lactobacillus acidophilus+Bifidobacterium infantis | Probiotic type |
| Johnston BC 2006 | 1 | 0.47 | 0.18-1.21 | NR | Lactobacillus acidophilus+Bifidobacterium infantis | Probiotic type |
| Szajewska H 2006 | 1 | 0.47 | 0.18-1.21 | NR | Lactobacillus acidophilus+Bifidobacterium infantis | Probiotic type |
| Guo Q 2019 | 1 | 0.47 | 0.18-1.21 | NR | Lactobacillus acidophilus+Bifidobacterium infantis | Probiotic type |
| Johnston BC 2006 | 1 | 0.96 | 0.61-1.50 | NR | Lactobacillus acidophilus+Lactobacillus bulgaricus | Probiotic type |
| Szajewska H 2006 | 1 | 0.96 | 0.61-1.50 | NR | Lactobacillus acidophilus+Lactobacillus bulgaricus | Probiotic type |
| Guo Q 2019 | 1 | 0.96 | 0.61-1.50 | NR | Lactobacillus acidophilus+Lactobacillus bulgaricus | Probiotic type |
| Goodman C 2021 | 7 | 0.6 | 0.37-0.97 | 66 | Lactobacillus bulgaricus | Probiotic type |
| Goodman C 2021 | 11 | 0.59 | 0.42-0.83 | 78 | Lactobacillus casei | Probiotic type |
| Johnston BC 2006 | 2 | 0.29 | 0.15-0.57 | 0 | Lactobacillus GG | Probiotic type |
| Videlock EJ 2012 | 8 | 0.40 | 0.20-0.81 | 67 | Lactobacillus GG | Probiotic type |
| Goodman C 2021 | 2 | 0.67 | 0.43-1.04 | 0 | Lactobacillus helveticus | Probiotic type |
| Goodman C 2021 | 3 | 0.6 | 0.41-0.88 | 24 | Lactobacillus paracasei | Probiotic type |
| Goodman C 2021 | 3 | 0.61 | 0.23-1.60 | 50 | Lactobacillus plantarum | Probiotic type |
| Fadin L 2023 | 2 | 0.95 | 0.51-1.77 | 0 | Lactobacillus reuteri | Probiotic type |
| Goodman C 2021 | 5 | 0.71 | 0.52-0.97 | 34 | Lactobacillus rhamnosus | Probiotic type |
| McFarland LV 2006 | 6 | 0.31 | 0.13-0.72 | NR | Lactobacillus rhamnosus GG | Probiotic type |
| Szajewska H 2006 | 2 | 0.29 | 0.15-0.57 | 0 | Lactobacillus rhamnosus GG | Probiotic type |
| Blaabjerg S 2017 | 2 | 0.29 | 0.15-0.57 | 0 | Lactobacillus rhamnosus GG | Probiotic type |
| Guo Q 2019 | 6 | 0.37 | 0.24-0.55 | 0 | Lactobacillus rhamnosus GG | Probiotic type |
| Fadin L 2023 | 4 | 0.29 | 0.17-0.49 | 0 | Lactobacillus rhamnosus GG | Probiotic type |
| Guo Q 2019 | 1 | 0.47 | 0.29-0.77 | NR | Lactobacillus sporogenes | Probiotic type |
| Johnston BC 2006 | 1 | 0.47 | 0.29-0.77 | NR | Lactobacillus sporogenes+fructo-oligosaccharides | Probiotic type |
| Liao W 2021 | 6 | 0.82 | 0.57-1.17 | 56 | Lactobacillus+Bifidobacterium | Probiotic type |
| Videlock EJ 2012 | 7 | 0.46 | 0.30-0.70 | 27 | Saccharomyces boulardii | Probiotic type |
| Hempel S 2012 | 15 | 0.48 | 0.35-0.65 | 56 | Saccharomyces | Probiotic type |
| Johnston BC 2006 | 1 | 0.19 | 0.07-0.55 | NR | Saccharomyces boulardii | Probiotic type |
| McFarland LV 2006 | 6 | 0.37 | 0.26-0.52 | NR | Saccharomyces boulardii | Probiotic type |
| Szajewska H 2006 | 1 | 0.19 | 0.07-0.55 | NR | Saccharomyces boulardii | Probiotic type |
| Jafarnejad S 2016 | 11 | 0.5 | 0.38-0.64 | 28 | Saccharomyces boulardii | Probiotic type |
| Blaabjerg S 2017 | 5 | 0.41 | 0.30-0.57 | 0 | Saccharomyces boulardii | Probiotic type |
| Guo Q 2019 | 9 | 0.36 | 0.24-0.54 | 76 | Saccharomyces boulardii | Probiotic type |
| Goodman C 2021 | 9 | 0.63 | 0.46-0.86 | 26 | Saccharomyces boulardii | Probiotic type |
| Liao W 2021 | 6 | 0.69 | 0.39-1.22 | 47 | Saccharomyces boulardii | Probiotic type |
| Szajewska H 2015 | 21 | 0.47 | 0.38-0.57 | 30 | Saccharomyces boulardii | Probiotic type |
| Goodman C 2021 | 6 | 0.62 | 0.37-1.04 | 69 | Saccharomyces thermophilus | Probiotic type |
| Videlock EJ 2012 | 10 | 0.62 | 0.46-0.84 | 52 | High RoB | Risk of bias |
| Jafarnejad S 2016 | 10 | 0.64 | 0.47-0.88 | 75 | High RoB | Risk of bias |
| Guo Q 2019 | 20 | 0.42 | 0.31-0.56 | 66 | High RoB | Risk of bias |
| Liao W 2021 | 5 | 0.45 | 0.27-0.76 | 0 | High RoB | Risk of bias |
| Goodman C 2021 | 36 | 0.59 | 0.50-0.70 | 54 | High/unclear RoB | Risk of bias |
| Liao W 2021 | 13 | 0.72 | 0.55-0.93 | 59 | Low RoB | Risk of bias |
| Videlock EJ 2012 | 14 | 0.48 | 0.35-0.68 | 51 | Low RoB | Risk of bias |
| Jafarnejad S 2016 | 11 | 0.61 | 0.45-0.84 | 33 | Low RoB | Risk of bias |
| Blaabjerg S 2017 | 3 | 0.36 | 0.08-1.64 | 82 | Low RoB | Risk of bias |
| Guo Q 2019 | 12 | 0.53 | 0.37-0.77 | 32 | Low RoB | Risk of bias |
| Goodman C 2021 | 6 | 0.78 | 0.57-1.07 | 65 | Low RoB | Risk of bias |
| Videlock EJ 2012 | 24 | 0.48 | 0.39-0.60 | 37 | Low/unclear RoB | Risk of bias |
| Goodman C 2021 | 29 | 0.65 | 0.54-0.78 | 57 | Low/unclear RoB | Risk of bias |
| Liao W 2021 | 18 | 0.57 | 0.42-0.77 | 63 | Unclear RoB | Risk of bias |
| Videlock EJ 2012 | 10 | 0.48 | 0.37-0.63 | 3 | Unclear RoB | Risk of bias |

**Note:** Values are subgroup-specific estimates reported by the included SRs/MAs. A single SR/MA could contribute more than one estimate to a subgroup and could contribute to more than one subgroup domain; subgroup comparisons are therefore across SRs/MAs and are exploratory.

**Abbreviations:** AAD, antibiotic-associated diarrhea; CFU, colony-forming units; CI, confidence interval; I², I-squared statistic; NR, not reported; RCT, randomized controlled trial; RoB, risk of bias; RR, risk ratio; SR/MA, systematic review/meta-analysis.

**Supplementary Table S5. Full-text articles excluded and reasons for exclusion**

| **Study ID** | **Reason for exclusion** |
| --- | --- |
| Huang JS 2002 | Did not report AAD-specific outcomes |
| Van Niel CW 2002 | Did not report AAD-specific outcomes |
| Mrukowicz JZ 2002 | Full text unavailable |
| McConaghy JR 2003 | Full text unavailable |
| Allen SJ 2004 | Did not report AAD-specific outcomes |
| Szajewska H 2005 | Not the most recent meta-analysis |
| Dendukuri N 2006 | No meta-analysis |
| Sazawal S 2006 | Did not report AAD-specific outcomes |
| Lewis S 2007 | No meta-analysis |
| Takahashi O 2007 | No meta-analysis |
| Johnston BC 2007 | Not the most recent meta-analysis |
| Szajewska H 2007 | Did not report AAD-specific outcomes |
| Szajewska H 2007 | Did not report AAD-specific outcomes |
| McFarland LV 2007 | Traveler's diarrhea |
| Chmielewska A 2008 | Did not report AAD-specific outcomes |
| Eren M 2010 | No meta-analysis |
| Bernaola Aponte G 2010 | Not the most recent meta-analysis |
| Allen SJ 2010 | Did not report AAD-specific outcomes |
| Johnston BC 2011 | Not the most recent meta-analysis |
| Szajewska H 2011 | Healthcare-associated diarrhea |
| Dinleyici EC 2012 | Did not report AAD-specific outcomes |
| Pan J 2012 | Did not report AAD-specific outcomes |
| Salari P 2012 | Did not report AAD-specific outcomes |
| Johnston BC 2012 | Clostridium difficile-associated diarrhea |
| Phavichitr N 2013 | No meta-analysis |
| Bernaola Aponte G 2013 | Did not report AAD-specific outcomes |
| Applegate JA 2013 | Did not report AAD-specific outcomes |
| Szajewska H 2013 | Did not report AAD-specific outcomes |
| van de Wetering FT 2013 | Protocol only |
| Goldenberg JZ 2013 | Clostridium difficile-associated diarrhea |
| Wu ZJ 2013 | Clostridium difficile-associated diarrhea |
| Suadoni MT 2014 | No meta-analysis |
| Urbanska M 2014 | No meta-analysis |
| Feizizadeh S 2014 | Did not report AAD-specific outcomes |
| Wanke M 2014 | Healthcare-associated diarrhea |
| Chunhong X 2015 | No meta-analysis |
| Patro-Golab B 2015 | Non-probiotic intervention |
| Goldenberg JZ 2015 | Not the most recent meta-analysis |
| Ahmadi E 2015 | Did not report AAD-specific outcomes |
| Vernaya M 2015 | Protocol only |
| Wong S 2015 | Protocol only |
| Wang YH 2016 | Chemoradiotherapy-induced diarrhea |
| Hayes SR 2016 | No meta-analysis |
| Lau CSM 2016 | Clostridium difficile-associated diarrhea |
| Sinclair A 2016 | Clostridium difficile-associated diarrhea |
| Liu MM 2017 | Chemoradiotherapy-induced diarrhea |
| Goldenberg JZ 2017 | Not the most recent meta-analysis |
| Goldenberg JZ 2017 | Clostridium difficile-associated diarrhea |
| Vernaya M 2017 | Clostridium difficile-associated diarrhea |
| Eberlin M 2018 | Non-probiotic intervention |
| Ianiro Gianluca 2018 | Did not report AAD-specific outcomes |
| Bae JM 2018 | Traveler's diarrhea |
| Devaraj NK 2019 | Chemoradiotherapy-induced diarrhea |
| Lu D 2019 | Chemoradiotherapy-induced diarrhea |
| Li YT 2019 | Did not report AAD-specific outcomes |
| Yang BMM 2019 | Did not report AAD-specific outcomes |
| McFarland LV 2019 | Traveler's diarrhea |
| Khosrodad N 2019 | Full text unavailable |
| Lin S 2020 | Chemoradiotherapy-induced diarrhea |
| Deng J 2020 | No meta-analysis |
| Bu F 2020 | Did not report AAD-specific outcomes |
| Collinson S 2020 | Did not report AAD-specific outcomes |
| Di JB 2020 | Did not report AAD-specific outcomes |
| Min L 2020 | Protocol only |
| Ma Y 2020 | Clostridium difficile-associated diarrhea |
| Long B 2021 | No meta-analysis |
| Xu X 2021 | No meta-analysis |
| Farahdina F 2021 | Non-probiotic intervention |
| Kambale RM 2021 | Did not report AAD-specific outcomes |
| Huang R 2021 | Did not report AAD-specific outcomes |
| Li Z 2021 | Did not report AAD-specific outcomes |
| Shimizu K 2021 | Did not report AAD-specific outcomes |
| Vassilopoulou L 2021 | Did not report AAD-specific outcomes |
| Wu HL 2021 | Did not report AAD-specific outcomes |
| Feng J 2022 | Chemoradiotherapy-induced diarrhea |
| Wei X 2022 | Non-probiotic intervention |
| Carona A 2022 | Did not report AAD-specific outcomes |
| Wang FJ 2022 | Did not report AAD-specific outcomes |
| Vecchio AL 2022 | Did not report AAD-specific outcomes |
| Wang Y 2022 | Did not report AAD-specific outcomes |
| Feng X 2022 | Protocol only |
| Feng XMM 2022 | Protocol only |
| Fan H 2022 | Traveler's diarrhea |
| Amiri Khosroshahi R 2023 | Chemoradiotherapy-induced diarrhea |
| Doar NW 2023 | No meta-analysis |
| Yang Q 2023 | No meta-analysis |
| Minaz Anmol 2024 | Did not report AAD-specific outcomes |
| Alharbi BF 2024 | Traveler's diarrhea |
| Fan H 2024 | Traveler's diarrhea |
| Siritientong T 2025 | Chemoradiotherapy-induced diarrhea |
| Yang M 2025 | Chemoradiotherapy-induced diarrhea |
| Zulaid A 2025 | Abstract only |
| Bagdadi B 2025 | Did not report AAD-specific outcomes |
| Chen L 2025 | Did not report AAD-specific outcomes |
| Wang Q 2025 | Did not report AAD-specific outcomes |
| Chen C 2025 | Did not report AAD-specific outcomes |
| McFarland LV 2025 | Did not report AAD-specific outcomes |
| Alao MA 2025 | Protocol only |
| Esmaeilinezhad Z 2025 | Clostridium difficile-associated diarrhea |
| Lyn Lee E 2025 | Full text unavailable |
| Szajewska H 2026 | Control group included probiotics |

**Abbreviation:** AAD, antibiotic-associated diarrhea.

**Supplementary Table S6. Item-level AMSTAR 2 assessments and overall methodological confidence ratings**

| Study ID | **Item 1** | **Item 2** | **Item 3** | **Item 4** | **Item 5** | **Item 6** | **Item 7** | **Item 8** | **Item 9** | **Item 10** | **Item 11** | **Item 12** | **Item 13** | **Item 14** | **Item 15** | **Item 16** | **Overall confidence** |
| --- | --- | --- | --- | --- | --- | --- | --- | --- | --- | --- | --- | --- | --- | --- | --- | --- | --- |
| Cremonini F 2002 | Y | N | Y | Y | Y | Y | N | PY | PY | N | Y | Y | Y | Y | Y | Y | Critically low |
| D'Souza AL 2002 | Y | N | Y | Y | Y | Y | N | Y | N | N | Y | N | Y | N | Y | Y | Critically low |
| Johnston BC 2006 | Y | N | Y | Y | Y | Y | N | Y | Y | N | Y | Y | Y | Y | Y | Y | Critically low |
| McFarland LV 2006 | Y | N | Y | Y | Y | Y | N | Y | Y | N | Y | Y | Y | Y | Y | Y | Critically low |
| Kale-Pradhan PB 2010 | Y | N | Y | Y | Y | Y | N | Y | Y | N | Y | Y | Y | Y | Y | Y | Critically low |
| Avadhani A 2011 | Y | N | Y | PY | Y | Y | Y | Y | Y | N | Y | Y | Y | Y | N | Y | Critically low |
| Pattani R 2013 | Y | N | Y | Y | Y | Y | N | Y | Y | N | Y | Y | Y | Y | Y | Y | Critically low |
| Xu HB 2017 | Y | N | Y | Y | Y | Y | N | Y | Y | N | Y | Y | Y | Y | Y | Y | Critically low |
| Goodman C 2021 | Y | N | Y | Y | Y | Y | N | Y | Y | N | Y | Y | Y | Y | Y | Y | Critically low |
| Liao W 2021 | Y | N | Y | PY | Y | Y | N | Y | Y | N | Y | Y | Y | Y | Y | Y | Critically low |
| Zhang L 2022 | Y | N | Y | PY | Y | Y | N | Y | Y | N | Y | Y | Y | Y | Y | Y | Critically low |
| Fadin L 2023 | Y | N | Y | PY | Y | Y | N | Y | Y | N | Y | Y | Y | Y | Y | Y | Critically low |
| Wanyama H 2025 | Y | N | Y | Y | Y | Y | N | Y | Y | N | Y | Y | Y | Y | Y | Y | Critically low |
| Hempel S 2012 | Y | Y | Y | Y | Y | Y | Y | Y | Y | N | Y | Y | Y | Y | Y | Y | High |
| Szajewska H 2006 | Y | N | Y | Y | Y | Y | Y | Y | Y | N | Y | Y | Y | Y | Y | Y | Low |
| Videlock EJ 2012 | Y | N | Y | Y | Y | Y | PY | Y | Y | N | Y | Y | Y | Y | Y | Y | Low |
| Szajewska H 2015 | Y | N | Y | Y | Y | Y | Y | Y | Y | N | Y | Y | Y | Y | Y | Y | Low |
| Jafarnejad S 2016 | Y | N | Y | Y | Y | Y | Y | Y | Y | N | Y | Y | Y | Y | Y | Y | Low |
| Blaabjerg S 2017 | Y | N | Y | PY | Y | Y | Y | Y | Y | N | Y | Y | Y | Y | Y | Y | Low |
| Cai J 2018 | Y | Y | Y | PY | Y | Y | N | Y | Y | N | Y | Y | Y | Y | Y | Y | Low |
| Guo Q 2019 | Y | N | Y | Y | Y | Y | Y | Y | Y | Y | Y | Y | Y | Y | Y | Y | Low |

**Note:** AMSTAR 2 items were rated as Y (Yes), PY (Partial Yes), or N (No). Overall methodological confidence was assigned according to the AMSTAR 2 guidance.

**Abbreviations:** AMSTAR 2, A MeaSurement Tool to Assess Systematic Reviews 2; N, No; PY, Partial Yes; SR/MA, systematic review/meta-analysis; Y, Yes.

**Supplementary Table S7. Primary-study-by-review citation matrix for overlap analysis**

| **Primary RCTs** | **Included SRs/MAs** | | | | | | | | | | | | | | | | | | | | | **Overlap summary** | |
| --- | --- | --- | --- | --- | --- | --- | --- | --- | --- | --- | --- | --- | --- | --- | --- | --- | --- | --- | --- | --- | --- | --- | --- |
| **Study ID** | Avadhani A 2011 | Blaabjerg S 2017 | Cai J 2018 | Cremonini F 2002 | D'Souza AL 2002 | Fadin L 2023 | Goodman C 2021 | Guo Q 2019 | Hempel S 2012 | Jafarnejad S 2016 | Johnston BC 2006 | Kale-Pradhan PB 2010 | Liao W 2021 | McFarland LV 2006 | Pattani R 2013 | Szajewska H 2006 | Szajewska H 2015 | Videlock EJ 2012 | Wanyama H 2025 | Xu HB 2017 | Zhang L 2022 | **No. of SRs/MAs including RCT** | **Included in >1 SR/MA?** |
| Adam J 1977 |  |  |  |  | 1 |  |  |  | 1 |  |  |  |  | 1 |  |  | 1 |  |  |  |  | 4 | Yes |
| Ahmad K 2013 |  |  | 1 |  |  |  |  | 1 |  |  |  |  |  |  |  |  |  |  |  |  |  | 2 | Yes |
| Ahuja MC 2002 |  |  |  |  |  |  |  |  |  |  |  | 1 |  |  |  |  |  |  |  |  |  | 1 | No |
| Alberda C 2018 |  |  |  |  |  |  |  |  |  |  |  |  |  |  |  |  |  |  | 1 |  |  | 1 | No |
| Allen SJ 2013 |  |  | 1 |  |  |  | 1 |  |  | 1 |  |  | 1 |  |  |  |  |  | 1 |  | 1 | 6 | Yes |
| Armuzzi A 2001 |  |  | 1 |  |  |  |  |  |  |  |  |  |  |  |  |  |  |  |  |  |  | 1 | No |
| Armuzzi AC 2001 |  |  |  | 1 |  |  |  |  |  | 1 |  | 1 | 1 | 1 |  |  |  | 1 |  |  |  | 6 | Yes |
| Arvola T 1999 |  | 1 | 1 | 1 |  | 1 | 1 |  | 1 |  | 1 | 1 |  | 1 |  | 1 |  | 1 |  |  |  | 11 | Yes |
| Barker AK 2017 |  |  |  |  |  |  |  |  |  |  |  |  |  |  |  |  |  |  | 1 |  |  | 1 | No |
| Beausoleil M 2007 |  |  | 1 |  |  |  | 1 |  | 1 | 1 |  | 1 | 1 |  | 1 |  |  | 1 |  |  | 1 | 9 | Yes |
| Bekar O 2010 |  |  |  |  |  |  |  |  |  |  |  |  | 1 |  |  |  |  |  |  |  |  | 1 | No |
| Benhamou PH 1999 |  |  |  |  |  |  |  | 1 | 1 |  |  |  |  |  |  |  |  |  |  |  |  | 2 | Yes |
| Beniwal RS 2003 | 1 |  |  |  |  |  | 1 |  |  |  |  |  |  |  |  |  |  |  |  |  |  | 2 | Yes |
| Bhalla A 2011 |  |  |  |  |  |  |  |  | 1 |  |  |  |  |  |  |  |  |  |  |  |  | 1 | No |
| Bin Z 2015 |  |  |  |  |  |  |  |  |  |  |  |  |  |  |  |  | 1 |  |  |  |  | 1 | No |
| Borgia M 1982 |  |  |  |  |  |  |  |  | 1 |  |  |  |  | 1 |  |  |  |  |  |  |  | 2 | Yes |
| Bravo MV 2008 |  |  | 1 |  |  |  | 1 |  | 1 |  |  |  | 1 |  |  |  | 1 |  |  |  |  | 5 | Yes |
| Can M 2006 | 1 |  | 1 |  |  |  | 1 |  | 1 | 1 |  |  | 1 |  | 1 |  | 1 | 1 |  |  |  | 9 | Yes |
| Casem RAO 2013 |  |  |  |  |  |  |  |  |  |  |  |  |  |  |  |  | 1 |  |  |  |  | 1 | No |
| Chatterjee S 2013 |  | 1 |  |  |  |  | 1 |  |  | 1 |  |  | 1 |  |  |  |  |  |  |  |  | 4 | Yes |
| Chotivitayatarakorn P 2017 |  |  |  |  |  |  |  |  |  |  |  |  | 1 |  |  |  |  |  |  |  |  | 1 | No |
| Chu Y 2012 |  |  |  |  |  |  |  |  |  |  |  |  |  |  |  |  | 1 |  |  |  |  | 1 | No |
| Cimperman L 2011 |  |  | 1 |  |  |  | 1 |  | 1 | 1 |  |  | 1 |  | 1 |  |  | 1 |  |  |  | 7 | Yes |
| Cindoruk M 2007 |  | 1 | 1 |  |  |  | 1 |  | 1 | 1 |  |  | 1 |  |  |  | 1 | 1 |  |  |  | 8 | Yes |
| Conway S 2007 |  | 1 | 1 |  |  | 1 |  | 1 |  |  |  |  |  | 1 |  |  |  |  |  |  |  | 5 | Yes |
| Correa NB 2005 |  |  | 1 |  |  |  |  | 1 | 1 |  |  |  |  | 1 |  | 1 |  | 1 |  |  |  | 6 | Yes |
| Cremonini F 2002 |  |  | 1 |  |  |  |  |  |  |  |  | 1 | 1 | 1 |  |  | 1 |  |  |  |  | 5 | Yes |
| Cremonini F 2002* |  |  |  |  |  |  |  |  |  | 1 |  |  |  |  |  |  |  | 1 |  |  |  | 2 | Yes |
| de Bortoli N 2007 |  |  |  |  |  |  |  |  | 1 |  |  |  |  |  |  |  |  |  |  |  |  | 1 | No |
| de Vrese M 2011 |  | 1 | 1 |  |  |  | 1 |  | 1 |  |  |  |  |  |  |  |  |  |  |  |  | 4 | Yes |
| Destura RV |  |  |  |  |  |  |  | 1 |  |  |  |  |  |  |  |  |  |  |  |  |  | 1 | No |
| Dharnai S 2017 |  |  |  |  |  |  |  | 1 |  |  |  |  |  |  |  |  |  |  |  |  |  | 1 | No |
| Dietrich CG 2014 |  |  | 1 |  |  |  |  |  |  |  |  |  |  |  |  |  |  |  |  |  |  | 1 | No |
| Duman DG 2005 |  | 1 | 1 |  |  |  | 1 |  | 1 |  |  |  |  |  |  |  | 1 |  |  |  |  | 5 | Yes |
| Ehrhardt S 2016 |  |  |  |  |  |  | 1 |  |  |  |  |  | 1 |  |  |  |  |  | 1 |  |  | 3 | Yes |
| Engelbrektso n 2009 |  |  | 1 |  |  |  |  |  | 1 |  |  |  |  |  |  |  |  |  |  |  |  | 2 | Yes |
| Erdeve O 2004 |  | 1 | 1 |  |  |  |  | 1 | 1 |  |  |  |  |  |  |  | 1 |  |  |  |  | 5 | Yes |
| Esposito C 2017 |  |  |  |  |  | 1 |  | 1 |  |  |  |  |  |  |  |  |  |  |  |  |  | 2 | Yes |
| Evans M 2016 |  |  |  |  |  |  | 1 |  |  |  |  |  | 1 |  |  |  |  |  | 1 |  |  | 3 | Yes |
| Felley CP 2001 |  |  |  |  |  |  |  |  | 1 |  |  |  |  |  |  |  |  |  |  |  |  | 1 | No |
| Feng SF 2008 |  |  |  |  |  |  |  |  |  |  |  |  |  |  |  |  |  |  |  | 1 |  | 1 | No |
| Fox MJ 2015 |  | 1 | 1 |  |  | 1 |  | 1 |  |  |  |  |  |  |  |  |  |  |  |  |  | 4 | Yes |
| Francavilla R 2014 |  |  |  |  |  |  |  |  |  |  |  |  | 1 |  |  |  |  |  |  |  |  | 1 | No |
| Frigerio G 1986 |  |  |  |  |  |  |  |  | 1 |  |  |  |  |  |  |  |  |  |  |  |  | 1 | No |
| Gan L 2008 |  |  |  |  |  |  |  |  |  |  |  |  |  |  |  |  |  |  |  | 1 |  | 1 | No |
| Diao ZY 2008 |  |  |  |  |  |  |  |  |  |  |  |  |  |  |  |  |  |  |  | 1 |  | 1 | No |
| Gao XW 2010 |  |  | 1 |  |  |  | 1 |  | 1 | 1 |  |  | 1 |  | 1 |  |  | 1 |  |  |  | 7 | Yes |
| Conway S 2007 |  |  |  |  |  |  |  |  | 1 |  |  |  |  |  |  |  |  |  |  |  |  | 1 | No |
| García Trallero O 2019 |  |  |  |  |  |  |  |  |  |  |  |  | 1 |  |  |  |  |  |  |  |  | 1 | No |
| Ge RH 2010 |  |  |  |  |  |  |  |  |  |  |  |  |  |  |  |  |  |  |  | 1 |  | 1 | No |
| Georgieva M 2015 |  |  |  |  |  | 1 |  | 1 |  |  |  |  |  |  |  |  |  |  |  |  |  | 2 | Yes |
| Gong CY 2011 |  |  |  |  |  |  |  |  |  |  |  |  |  |  |  |  |  |  |  | 1 |  | 1 | No |
| Gotz V 1979 |  |  |  | 1 | 1 |  | 1 |  | 1 | 1 |  | 1 |  | 1 | 1 |  |  | 1 |  |  |  | 9 | Yes |
| Haghdoost M 2017 |  |  |  |  |  |  |  |  |  |  |  |  | 1 |  |  |  |  |  |  |  |  | 1 | No |
| He LY 2009 |  |  |  |  |  |  |  |  |  |  |  |  |  |  |  |  |  |  |  | 1 |  | 1 | No |
| Helps A 2015 |  |  |  |  |  |  | 1 |  |  |  |  |  | 1 |  |  |  |  |  | 1 |  |  | 3 | Yes |
| Hickson M 2007 |  |  | 1 |  |  |  | 1 |  | 1 | 1 |  |  | 1 |  | 1 |  |  | 1 |  |  | 1 | 8 | Yes |
| Hickson M 2007* | 1 |  |  |  |  |  |  |  |  |  |  |  |  |  |  |  |  |  |  |  |  | 1 | No |
| Hong ZY 2013 |  |  |  |  |  |  |  |  |  |  |  |  |  |  |  |  |  |  |  | 1 |  | 1 | No |
| Horosheva T 2014 |  |  |  |  |  |  | 1 |  |  |  |  |  |  |  |  |  |  |  |  |  |  | 1 | No |
| Huang LH 2011 |  |  |  |  |  |  |  |  |  |  |  |  |  |  |  |  |  |  |  | 1 |  | 1 | No |
| Hurduc V 2009 |  |  | 1 |  |  |  |  |  |  |  |  |  |  |  |  |  |  |  |  |  |  | 1 | No |
| Iamharit J 2010 |  |  |  |  |  |  | 1 |  |  |  |  |  |  |  |  |  |  |  |  |  |  | 1 | No |
| Imase K 2008 |  | 1 |  |  |  |  | 1 |  |  |  |  |  |  |  |  |  |  |  |  |  |  | 2 | Yes |
| Jiang L 2018 |  |  |  |  |  |  |  |  |  |  |  |  | 1 |  |  |  |  |  |  |  |  | 1 | No |
| Jindal M 2017 |  |  |  |  |  |  |  | 1 |  |  |  |  |  |  |  |  |  |  |  |  |  | 1 | No |
| Jirapinyo P 2002 |  |  |  |  |  |  |  | 1 | 1 |  | 1 |  |  | 1 |  | 1 |  | 1 |  |  |  | 6 | Yes |
| Ke DH 2009 |  |  |  |  |  |  |  |  |  |  |  |  |  |  |  |  |  |  |  | 1 |  | 1 | No |
| Khanal A 2015 |  |  |  |  |  |  | 1 |  |  |  |  |  |  |  |  |  |  |  |  |  |  | 1 | No |
| Kim MN 2008 |  | 1 | 1 |  |  |  |  |  | 1 |  |  |  |  |  |  |  |  |  |  |  |  | 3 | Yes |
| King S 2010 |  |  |  |  |  |  |  | 1 |  |  |  |  |  |  |  |  |  |  |  |  |  | 1 | No |
| Kolodziej M 2019 |  |  |  |  |  | 1 |  | 1 |  |  |  |  |  |  |  |  |  |  |  |  |  | 2 | Yes |
| Koning CJ 2008 |  |  | 1 |  |  |  | 1 |  | 1 | 1 |  |  | 1 |  |  |  |  | 1 |  |  |  | 6 | Yes |
| Koning CJ 2010 |  |  | 1 |  |  |  |  |  | 1 | 1 |  |  |  |  |  |  |  | 1 |  |  |  | 4 | Yes |
| Kotowska M 2005 |  |  | 1 |  |  | 1 |  | 1 | 1 |  | 1 |  |  | 1 |  | 1 | 1 | 1 |  |  |  | 9 | Yes |
| Kyriakos N 2013 |  |  |  |  |  |  |  |  |  |  |  |  |  |  |  |  | 1 |  |  |  |  | 1 | No |
| La Rosa M 2003 |  |  |  |  |  |  |  | 1 | 1 |  | 1 |  |  | 1 |  |  |  | 1 |  |  |  | 5 | Yes |
| Lewis SJ 1998 | 1 |  |  | 1 | 1 |  | 1 |  | 1 |  |  |  |  | 1 | 1 |  | 1 | 1 |  |  | 1 | 10 | Yes |
| Li D 2010 |  |  |  |  |  |  |  |  | 1 |  |  |  |  |  |  |  |  |  |  |  |  | 1 | No |
| Li HZ 2012 |  |  |  |  |  |  |  |  |  |  |  |  |  |  |  |  |  |  |  | 1 |  | 1 | No |
| Lighthouse J 2004 |  |  |  |  |  |  |  |  | 1 |  |  |  |  |  |  |  |  |  |  |  |  | 1 | No |
| Ligny G 1976 |  |  |  |  |  |  |  |  | 1 |  |  |  |  |  |  |  |  |  |  |  |  | 1 | No |
| Lionetti E 2006 |  |  | 1 |  |  |  |  |  |  |  |  |  |  |  |  |  |  |  |  |  |  | 1 | No |
| Liu AL 2007 |  |  |  |  |  |  |  |  |  |  |  |  |  |  |  |  |  |  |  | 1 |  | 1 | No |
| Liu J 2008 |  |  |  |  |  |  |  |  |  |  |  |  |  |  |  |  |  |  |  | 1 |  | 1 | No |
| Liu QF 2008 |  |  |  |  |  |  |  |  |  |  |  |  |  |  |  |  |  |  |  | 1 |  | 1 | No |
| Lonnermark E 2010 |  |  | 1 |  |  |  | 1 |  | 1 | 1 |  |  | 1 |  |  |  |  | 1 |  |  |  | 6 | Yes |
| Lou JX 2006 |  |  |  |  |  |  |  |  |  |  |  |  |  |  |  |  |  |  |  | 1 |  | 1 | No |
| Ma FF 2009 |  |  |  |  |  |  |  |  |  |  |  |  |  |  |  |  |  |  |  | 1 |  | 1 | No |
| Manfredi M 2012 |  |  |  |  |  |  |  |  |  |  |  |  | 1 |  |  |  |  |  |  |  |  | 1 | No |
| McFarland LV 1995 | 1 |  |  | 1 | 1 |  | 1 |  | 1 | 1 |  |  |  | 1 | 1 |  | 1 | 1 |  |  |  | 10 | Yes |
| McFarland LV 1994 |  |  |  |  |  |  |  |  |  |  |  |  |  | 1 |  |  |  |  |  |  |  | 1 | No |
| Meng YL 2012 |  |  |  |  |  |  |  |  |  |  |  |  |  |  |  |  |  |  |  | 1 |  | 1 | No |
| Merenstein DJ 2009 |  | 1 | 1 |  |  | 1 |  | 1 | 1 |  |  |  |  |  |  |  |  |  |  |  |  | 5 | Yes |
| Monteiro E 1981 |  |  |  |  |  |  |  |  | 1 |  |  |  |  |  |  |  | 1 |  |  |  |  | 2 | Yes |
| Morrow LE 2010 |  |  | 1 |  |  |  |  |  |  |  |  |  |  |  |  |  |  |  |  |  |  | 1 | No |
| Myllyluoma E 2005 |  |  | 1 |  |  |  |  |  | 1 | 1 |  |  |  |  |  |  |  | 1 |  |  |  | 4 | Yes |
| Nista EC 2004 |  |  | 1 |  |  |  |  |  |  | 1 |  |  | 1 | 1 |  |  |  | 1 |  |  |  | 5 | Yes |
| Ojetti V 2012 |  | 1 |  |  |  |  |  |  |  |  |  |  |  |  |  |  |  |  |  |  |  | 1 | No |
| Olek A 2017 |  | 1 |  |  |  | 1 |  | 1 |  |  |  |  |  |  |  |  |  |  |  |  |  | 3 | Yes |
| Orrhage K 1994 |  |  |  |  | 1 |  |  |  |  | 1 |  |  |  | 1 |  |  |  | 1 |  |  |  | 4 | Yes |
| Ouwehand AC 2014 |  |  | 1 |  |  |  | 1 |  |  | 1 |  |  | 1 |  |  |  |  |  | 1 |  |  | 5 | Yes |
| Padilla Ruiz M 2013 |  |  |  |  |  |  |  |  |  |  |  |  | 1 |  |  |  |  |  |  |  |  | 1 | No |
| Park SK 2007 |  | 1 |  |  |  |  |  |  | 1 |  |  |  |  |  |  |  |  |  |  |  |  | 2 | Yes |
| Peng F 2014 |  |  |  |  |  |  |  | 1 |  |  |  |  |  |  |  |  |  |  |  |  |  | 1 | No |
| Pirker A 2013 |  |  |  |  |  |  | 1 |  |  |  |  |  |  |  |  |  |  |  | 1 |  |  | 2 | Yes |
| Plomer M 2020 |  |  |  |  |  |  | 1 |  |  |  |  |  |  |  |  |  |  |  |  |  |  | 1 | No |
| Plummer S 2004 | 1 |  | 1 |  |  |  | 1 |  | 1 |  |  |  |  |  | 1 |  |  |  |  |  |  | 5 | Yes |
| Popescu A 2020 |  |  |  |  |  |  |  |  |  |  |  |  |  |  |  |  |  |  | 1 |  |  | 1 | No |
| Pozzoni P 2012 |  |  | 1 |  |  |  | 1 |  |  | 1 |  |  | 1 |  | 1 |  | 1 |  |  |  | 1 | 7 | Yes |
| Rajkumar C 2020 |  |  |  |  |  |  | 1 |  |  |  |  |  | 1 |  |  |  |  |  | 1 |  | 1 | 4 | Yes |
| Reid G 1992 |  |  |  |  |  |  |  |  | 1 |  |  |  |  |  |  |  |  |  |  |  |  | 1 | No |
| Romeo A 2019 |  |  |  |  |  |  |  |  |  |  |  |  | 1 |  |  |  |  |  |  |  |  | 1 | No |
| Ruszczyński M 2008 |  |  | 1 |  |  | 1 |  | 1 | 1 |  |  | 1 |  |  |  |  |  | 1 |  |  |  | 6 | Yes |
| Safdar N 2008 |  |  | 1 |  |  |  | 1 |  | 1 | 1 |  |  |  |  |  |  |  | 1 |  |  | 1 | 6 | Yes |
| Sampalis J 2010 |  |  | 1 |  |  |  | 1 |  | 1 | 1 |  |  |  |  | 1 |  |  | 1 | 1 |  |  | 7 | Yes |
| Saneeyan H 2011 |  |  |  |  |  |  |  | 1 | 1 |  |  |  |  |  |  |  |  |  |  |  |  | 2 | Yes |
| Schrezenmeir J 2004 |  |  |  |  |  |  |  |  | 1 |  |  |  |  |  |  |  |  |  |  |  |  | 1 | No |
| Seki H 2003 |  |  |  |  |  |  |  |  |  |  |  |  |  | 1 |  |  |  |  |  |  |  | 1 | No |
| Selinger CP 2013 |  |  |  |  |  |  | 1 |  | 1 | 1 |  |  | 1 |  |  |  |  |  | 1 |  |  | 5 | Yes |
| Shafaghi A 2016 |  |  |  |  |  |  |  |  |  |  |  |  | 1 |  |  |  |  |  |  |  |  | 1 | No |
| Shan LS 2013 |  |  | 1 |  |  |  |  | 1 |  |  |  |  |  |  |  |  | 1 |  |  |  |  | 3 | Yes |
| Shao Y 2011 |  |  |  |  |  |  |  |  |  |  |  |  |  |  |  |  |  |  |  | 1 |  | 1 | No |
| Shavakhi A 2013 |  |  |  |  |  |  |  |  |  |  |  |  | 1 |  |  |  |  |  |  |  |  | 1 | No |
| Sheu 2002 |  |  | 1 |  |  |  |  |  | 1 |  |  |  |  |  |  |  |  |  |  |  |  | 2 | Yes |
| Shimbo I 2005 |  |  |  |  |  |  | 1 |  |  |  |  |  |  |  |  |  |  |  |  |  |  | 1 | No |
| Song HJ 2010 |  |  | 1 |  |  |  | 1 |  | 1 | 1 |  |  | 1 |  | 1 |  |  | 1 | 1 |  |  | 8 | Yes |
| Song MJ 2010 |  |  |  |  |  |  |  |  | 1 |  |  |  |  |  |  |  | 1 |  |  |  |  | 2 | Yes |
| Souza DN 2012 |  |  | 1 |  |  |  |  |  |  | 1 |  |  |  |  |  |  |  |  |  |  |  | 2 | Yes |
| Stein GY 2007 |  |  |  |  |  |  |  |  | 1 |  |  |  |  |  |  |  |  |  |  |  |  | 1 | No |
| Sullivan 2003 |  |  | 1 |  |  |  |  |  | 1 |  |  |  |  |  |  |  |  |  |  |  |  | 2 | Yes |
| Sunenshine RH 2006 | 1 |  |  |  |  |  |  |  |  |  |  |  |  |  |  |  |  |  |  |  |  | 1 | No |
| Surawicz CM 1989 |  |  |  | 1 | 1 |  | 1 |  | 1 | 1 |  |  |  | 1 | 1 |  | 1 | 1 |  |  |  | 9 | Yes |
| Sykora J 2005 |  |  | 1 |  |  |  |  | 1 |  |  |  |  |  |  |  |  |  |  |  |  |  | 2 | Yes |
| Szajewska H 2009 |  |  | 1 |  |  |  |  | 1 | 1 |  |  |  |  | 1 |  |  |  | 1 |  |  |  | 5 | Yes |
| Szajewska H 2001 |  |  |  |  |  |  |  |  |  |  |  |  |  | 1 |  |  |  |  |  |  |  | 1 | No |
| Szymanski H 2008 |  |  | 1 |  |  | 1 |  | 1 | 1 |  |  |  |  |  |  |  |  | 1 |  |  |  | 5 | Yes |
| Tang J 2013 |  |  |  |  |  |  |  |  |  |  |  |  |  |  |  |  |  |  |  | 1 |  | 1 | No |
| Tankanow RM 1990 |  | 1 |  |  | 1 |  |  | 1 | 1 |  | 1 | 1 |  | 1 |  | 1 |  | 1 |  |  |  | 9 | Yes |
| Thomas MR 2001 | 1 |  | 1 |  |  |  | 1 |  | 1 | 1 |  | 1 | 1 | 1 | 1 |  |  | 1 |  |  |  | 10 | Yes |
| Tong FM 2012 |  |  |  |  |  |  |  |  |  |  |  |  |  |  |  |  |  |  |  | 1 |  | 1 | No |
| Tursi A 2004 |  |  | 1 |  |  |  |  |  | 1 |  |  |  |  |  |  |  |  |  |  |  |  | 2 | Yes |
| Vanderhoof JA1999 |  | 1 | 1 | 1 | 1 | 1 |  | 1 | 1 |  | 1 | 1 |  | 1 |  | 1 |  | 1 |  |  |  | 12 | Yes |
| Velasco M 2019 |  |  |  |  |  |  | 1 |  |  |  |  |  |  |  |  |  |  |  |  |  |  | 1 | No |
| Wan CM 2017 |  |  |  |  |  |  |  | 1 |  |  |  |  |  |  |  |  |  |  |  |  |  | 1 | No |
| Wang CX 2012 |  |  |  |  |  |  |  |  |  |  |  |  |  |  |  |  |  |  |  | 1 |  | 1 | No |
| Wang Y 2012 |  |  |  |  |  |  |  |  |  |  |  |  |  |  |  |  |  |  |  | 1 |  | 1 | No |
| Wenus C 2008 |  |  | 1 |  |  |  | 1 |  | 1 | 1 |  |  | 1 |  | 1 |  |  | 1 |  |  |  | 7 | Yes |
| Witsell DL 1995 |  |  |  |  |  |  |  |  |  |  |  |  |  | 1 |  |  |  |  |  |  |  | 1 | No |
| Wong S 2014 |  |  |  |  |  |  | 1 |  |  | 1 |  |  |  |  |  |  |  |  |  |  |  | 2 | Yes |
| Wong SS 2013 |  |  |  |  |  |  |  |  |  |  |  |  |  |  |  |  |  |  | 1 |  |  | 1 | No |
| Wright K 2015 |  |  |  |  |  |  |  | 1 |  | 1 |  |  | 1 |  |  |  |  |  |  |  | 1 | 4 | Yes |
| Wu J 2020 |  |  |  |  |  |  | 1 |  |  |  |  |  |  |  |  |  |  |  | 1 |  |  | 2 | Yes |
| Wunderlich PF 1989 |  |  |  |  | 1 |  |  |  | 1 |  |  |  |  | 1 | 1 |  |  | 1 |  |  |  | 5 | Yes |
| Xi XY 2012 |  |  |  |  |  |  |  |  |  |  |  |  |  |  |  |  |  |  |  | 1 |  | 1 | No |
| Xie DY 2004 |  |  |  |  |  |  |  |  |  |  |  |  |  |  |  |  |  |  |  | 1 |  | 1 | No |
| Xu XQ 2013 |  |  |  |  |  |  |  |  |  |  |  |  |  |  |  |  |  |  |  | 1 |  | 1 | No |
| Xu YH 2011 |  |  |  |  |  |  |  |  |  |  |  |  |  |  |  |  |  |  |  | 1 |  | 1 | No |
| Yan J 2007 |  |  |  |  |  |  |  |  |  |  |  |  |  |  |  |  |  |  |  | 1 |  | 1 | No |
| Yao BH 2011 |  |  |  |  |  |  |  |  |  |  |  |  |  |  |  |  |  |  |  | 1 |  | 1 | No |
| Yasar B 2010 |  |  |  |  |  |  |  |  | 1 |  |  |  |  |  |  |  |  |  |  |  |  | 1 | No |
| Yoon H 2011 |  |  | 1 |  |  |  |  |  | 1 |  |  |  |  |  |  |  |  |  |  |  |  | 2 | Yes |
| Zakordonets L 2016 |  |  |  |  |  |  |  | 1 |  |  |  |  |  |  |  |  |  |  |  |  |  | 1 | No |
| Zhang B 2015 |  |  |  |  |  |  |  | 1 |  |  |  |  |  |  |  |  |  |  |  |  |  | 1 | No |
| Zhao HM2014 |  |  |  |  |  |  |  | 1 |  |  |  |  |  |  |  |  | 1 |  |  |  |  | 2 | Yes |
| Zhao L 2006 |  |  |  |  |  |  |  |  |  |  |  |  |  |  |  |  |  |  |  | 1 |  | 1 | No |
| Zheng YJ 2012 |  |  | 1 |  |  |  |  | 1 |  |  |  |  |  |  |  |  |  |  |  |  |  | 2 | Yes |
| Zhou L 2007 |  |  |  |  |  |  |  |  |  |  |  |  |  |  |  |  |  |  |  | 1 |  | 1 | No |
| Zhu XL 2012 |  |  |  |  |  |  |  |  |  |  |  |  |  |  |  |  |  |  |  | 1 |  | 1 | No |
| Zojaji H 2013 |  | 1 |  |  |  |  |  |  |  |  |  |  |  |  |  |  | 1 |  |  |  |  | 2 | Yes |

**Note:** A value of 1 indicates that a primary RCT was included in the corresponding SR/MA; a blank cell indicates that it was not included. The final two columns summarize how many included SRs/MAs contained each RCT and whether that RCT appeared in more than one SR/MA. This matrix was used for the primary-study overlap analyses.

**Abbreviations:** CCA, corrected covered area; RCT, randomized controlled trial; SR/MA, systematic review/meta-analysis.

**Supplementary Table S8A. Sensitivity analyses of the primary RR synthesis**

| **Sensitivity analysis** | **SRs/MAs included (n)** | **Pooled RR (95% CI)** | **P value for overall effect** | **I² (%)** | **P value for heterogeneity** |
| --- | --- | --- | --- | --- | --- |
| Unrestricted primary RR analysis | 18 | 0.543 (0.480-0.615) | <0.001 | 77.8 | <0.001 |
| Restricted to SRs/MAs with pairwise CCA <15% | 6 | 0.595 (0.449-0.788) | <0.001 | 87.8 | <0.001 |
| Restricted to SRs/MAs with pairwise CCA <10% | 5 | 0.633 (0.469-0.853) | 0.003 | 88.2 | <0.001 |
| Restricted to SRs/MAs with high or low AMSTAR 2 confidence | 7 | 0.524 (0.483-0.569) | <0.001 | 3.3 | 0.401 |

**Note:** The unrestricted analysis included all 18 SRs/MAs reporting RR estimates. For overlap-restricted analyses, reviews were ranked by AMSTAR 2 methodological confidence, then by number of included RCTs, and then by publication year (latest first). Reviews were considered sequentially and retained only if the pairwise CCA with every previously retained review was below the specified threshold (<15% or <10%). OR-reporting reviews were not included in these RR-specific sensitivity analyses. The methodological-confidence analysis was restricted to SRs/MAs rated high or low by AMSTAR 2.

**Abbreviations:** AMSTAR 2, A MeaSurement Tool to Assess Systematic Reviews 2; CCA, corrected covered area; CI, confidence interval; OR, odds ratio; RCT, randomized controlled trial; RR, risk ratio; SR/MA, systematic review/meta-analysis.

**Supplementary Table S8B. Leave-one-review-out sensitivity analysis of the primary RR synthesis**

| **SR/MA omitted** | **SRs/MAs included (n)** | **Pooled RR (95% CI)** | **P value for overall effect** | **I² (%)** | **P value for heterogeneity** |
| --- | --- | --- | --- | --- | --- |
| McFarland LV 2006 | 17 | 0.551 (0.486-0.625) | <0.001 | 77.9 | <0.001 |
| Cremonini F 2002 | 17 | 0.552 (0.488-0.626) | <0.001 | 77.8 | <0.001 |
| Kale-Pradhan PB 2010 | 17 | 0.550 (0.486-0.623) | <0.001 | 78.4 | <0.001 |
| Wanyama H 2025 | 17 | 0.540 (0.474-0.614) | <0.001 | 79.1 | <0.001 |
| Johnston BC 2006 | 17 | 0.547 (0.483-0.621) | <0.001 | 78.8 | <0.001 |
| Szajewska H 2006 | 17 | 0.547 (0.482-0.620) | <0.001 | 78.8 | <0.001 |
| Fadin L 2023 | 17 | 0.546 (0.481-0.619) | <0.001 | 78.9 | <0.001 |
| Goodman C 2021 | 17 | 0.535 (0.468-0.613) | <0.001 | 78.9 | <0.001 |
| Liao W 2021 | 17 | 0.537 (0.470-0.613) | <0.001 | 79.0 | <0.001 |
| Jafarnejad S 2016 | 17 | 0.540 (0.472-0.616) | <0.001 | 79.1 | <0.001 |
| Blaabjerg S 2017 | 17 | 0.546 (0.481-0.621) | <0.001 | 78.7 | <0.001 |
| Guo Q 2019 | 17 | 0.551 (0.485-0.625) | <0.001 | 77.3 | <0.001 |
| Hempel S 2012 | 17 | 0.539 (0.470-0.617) | <0.001 | 79.1 | <0.001 |
| Zhang L 2022 | 17 | 0.537 (0.500-0.576) | <0.001 | 25.9 | 0.157 |
| Videlock EJ 2012 | 17 | 0.543 (0.476-0.620) | <0.001 | 78.7 | <0.001 |
| Pattani R 2013 | 17 | 0.538 (0.473-0.614) | <0.001 | 79.1 | <0.001 |
| Szajewska H 2015 | 17 | 0.549 (0.483-0.624) | <0.001 | 77.6 | <0.001 |
| Avadhani A 2011 | 17 | 0.541 (0.475-0.617) | <0.001 | 79.1 | <0.001 |

**Note:** Each RR-reporting SR/MA was sequentially omitted, and the DerSimonian-Laird random-effects model was refitted using the remaining 17 reviews. The unrestricted primary RR synthesis included 18 reviews (RR 0.543, 95% CI 0.480-0.615; I² = 77.8%). Values are shown to three decimal places to preserve the leave-one-review-out results.

**Abbreviations:** CI, confidence interval; I², I-squared statistic; RR, risk ratio; SR/MA, systematic review/meta-analysis.

**Supplementary Figure S1. Forest plot of the primary RR synthesis restricted to SRs/MAs with high or low AMSTAR 2 methodological confidence**

**Abbreviations:** AMSTAR 2, A MeaSurement Tool to Assess Systematic Reviews 2; CI, confidence interval; DL, DerSimonian-Laird; I², I-squared statistic; RR, risk ratio; SR/MA, systematic review/meta-analysis.

**Supplementary Figure S2. Forest plot of the primary RR synthesis restricted to a low-overlap subset with all pairwise CCA values <15%**

**Abbreviations:** CCA, corrected covered area; CI, confidence interval; DL, DerSimonian-Laird; I², I-squared statistic; RR, risk ratio; SR/MA, systematic review/meta-analysis.

**Supplementary Figure S3. Forest plot of the primary RR synthesis restricted to a low-overlap subset with all pairwise CCA values <10%**

**Abbreviations:** CCA, corrected covered area; CI, confidence interval; DL, DerSimonian-Laird; I², I-squared statistic; RR, risk ratio; SR/MA, systematic review/meta-analysis.
